# High-precision detection and navigation surgery of colorectal cancer micrometastases

**DOI:** 10.1186/s12951-023-02171-z

**Published:** 2023-11-02

**Authors:** Shengjie Ma, Bin Sun, Mengfei Li, Tianyang Han, Chenlong Yu, Xin Wang, Xue Zheng, Shuang Li, Shoujun Zhu, Quan Wang

**Affiliations:** 1 Department of Gastrocolorectal Surgery, General Surgery Center, The First Hospital of Jilin University, Changchun, 130012 People’s Republic of China; 2 Joint Laboratory of Opto-Functional Theranostics in Medicine and Chemistry, The First Hospital of Jilin University, Changchun, 130021 People’s Republic of China; 3 State Key Laboratory of Supramolecular Structure and Materials, College of Chemistry, Jilin University, Changchun, 130012 People’s Republic of China

**Keywords:** Organic NIR-II fluorophores, NIR-II fluorescence-guided surgery, Lymphatic metastasis, Peritoneal metastasis, Colorectal cancer

## Abstract

Surgical resection is an effective treatment for colorectal cancer (CRC) patients, whereas occult metastases hinder the curative effect. Currently, there is no effective method to achieve intraoperatively diagnosis of tumor-positive lymph nodes (LNs). Herein, we adopt a near-infrared-II (NIR-II) organic donor-pi-acceptor-pi-donor probe FE-2PEG, which exhibits bright fluorescence over 1100 nm, excellent photostability, blood circulation time, and biocompatibility, to achieve high-performance bioimaging with improved temporal and spatial resolution. Importantly, the FE-2PEG shows efficient passive enrichment in orthotopic CRC, metastatic mesenteric LNs, and peritoneal metastases by enhanced permeability and retention effect. Under NIR-II fluorescence-guided surgery (FGS), the peritoneal micrometastases were resected with a sensitivity of 94.51%, specificity of 86.59%, positive predictive value (PPV) of 96.57%, and negative predictive value of 79.78%. The PPV still achieves 96.07% even for micrometastases less than 3 mm. Pathological staining and NIR-II microscopy imaging proved that FE-2PEG could successfully delineate the boundary between the tumor and normal tissues. Dual-color NIR-II imaging strategy with FE-2PEG (1100 ~ 1300 nm) and PbS@CdS quantum dots (> 1500 nm) successfully protects both blood supply and normal tissues during surgery. The NIR-II-based FGS provides a promising prospect for precise intraoperative diagnosis and minimally invasive surgery of CRC.

**Supplementary Information:**

The online version contains supplementary material available at 10.1186/s12951-023-02171-z.

## Introduction

Colorectal cancer (CRC) is the third most common cancer and is one of the leading causes of cancer-related deaths worldwide [1, 2]. Surgical resection, a traditional yet effective cancer therapy, is considered the optimal choice for CRC patients. Surgeons have historically relied on anatomical localization, palpation feedback, and clinical experience to achieve microscopic tumor-free margins (R0 resection). However, according to a previous report, higher positive surgical margin (PSM) rates contribute to higher T-category, with T4 CRC having high PSM rates up to 27% [3]. Complete surgical R0 resection is associated with lower recurrence rates and better overall survival [4, 5]. Nevertheless, R0 resection is still a challenge for many patients, particularly with potential lymphatic metastasis and peritoneal dissemination. Complete removal of the primary tumor, draining lymph nodes (LNs), and extra-regional metastatic nodules are theoretically necessary to prevent postoperative recurrence and metastasis caused by tumor dissemination. Cytoreductive surgery combined with hyperthermic intraperitoneal chemotherapy is a treatment option for CRC patients with peritoneal metastasis (PM) [6, 7]. MRI, CT, and X-rays, with their associated hazard of radiation, have been used for diagnosis and surgical planning, but they lack real-time intraoperative surgical navigation. Therefore, real-time fluorescence-guided surgery (FGS) has tremendous advantages in achieving precise intraoperative diagnosis and surgical navigation of CRC.

Convectional FGS is mainly performed in the first near-infrared window (NIR-I, 700 ~ 900 nm), which is limited by shallow tissue penetration (~ 1 mm) and unsatisfactory signal-to-background ratio (SBR) by tissue autofluorescence and photon scattering [8–10]. The second near-infrared window (NIR-II, > 900 nm) has shown promising results for both preoperative imaging and intraoperative navigation, receiving increased attention in recent years. Imaging in the NIR-II window can significantly overcome the effects of strong tissue absorption, autofluorescence, and photon scattering, offering advantages such as micron-scale spatial resolution, higher tumor-to-normal tissue ratio (TNR), and deeper tissue penetration (~ 20 mm) [11–14]. NIR-II FGS utilizes imaging probes to non-invasively depict the tumor margins with high temporal-spatial resolution in real-time. In addition, small and portable NIR-II FGS systems can be integrated with the laparoscopy or Da Vinci robotic system to assist in the operation. Overall, NIR-II FGS could reduce the reliance on non-specific visual cues, leading to better surgical resection and fewer surgery-related complications. More importantly, NIR-II FGS can illuminate and excise occult and microscopic metastatic lesions, reducing postoperative tumor recurrence.

Near-infrared tracers used in intraoperative imaging can favor precise preoperative delineation of tumor margins, intraoperative minimal residual disease detection, and postoperative verification of complete resection [10, 15]. Therefore, the development of a high-quality NIR-II fluorescent tracer is essential for the advancement of NIR-II FGS. Currently, clinically available fluorescent probes approved by the U.S. Food and Drug Administration include indocyanine green (ICG), methylene blue, and 5-aminolevulinic acid. ICG has been widely used as a NIR-I tracer to assist in radical LNs dissection [16, 17], detection of colorectal liver metastases [18–20], and evaluation of colon perfusion [21–23]. ICG is also suitable for NIR-II fluorescence imaging because its emission tail extends into the NIR-II region with relatively reasonable brightness, even though its emission peak is not centered in the NIR-II region [24]. However, ICG has some drawbacks, such as a short blood circulation time and being more prone to fluorescence quenching [25, 26].

For lab-scale small animal imaging, IR-780 has enabled multifunctional applications, including sentinel lymph node mapping, NIR imaging for tumor detection, and cancer cell navigation during surgery [15, 27, 28]. Serum albumin with long circulation time has the ability to encapsulate hydrophobic dye IR-780 through hydrophobic pockets and improve nanoparticle stability [29, 30]. Coating IR-780 with exogenous or endogenous albumin could increase the blood circulation half-time and hence the tumor uptake [31]. Although bovine serum albumin (BSA) @IR-780 has the potential prospect for clinical application, the unsatisfactory TNR resulting from tissue absorption fluorescence is still problematic. Many fluorescent probes have been designed for NIR-II imaging, such as organic molecule probes [32, 33], single-walled carbon nanotubes [34], quantum dots (QDs) [35–37], and rare-earth doped nanoparticles [38, 39]. Among these probes, NIR-II organic donor-pi-acceptor-pi-donor (D-A-D) fluorophores have broad clinical translational prospects due to their excellent biocompatibility, photostability, and passive accumulation based on enhanced permeability and retention (EPR) effect [32]. Functionalization of the D-A-D probes with poly (ethylene glycol) (PEG) is the classic strategy employed to improve their blood circulation time, which can achieve high tumor uptake [40, 41]. Activable fluorescent probes can turn on fluorescence by reacting with acceptors in the cellar surface [42, 43] or specific stimuli in the tumor microenvironment of CRC, such as enzymes [44–50], acidity [51], hydrogen peroxide [52], glutathione [53], and hydrogen sulfide [54]. On the one hand, activable fluorescent probes can obtain higher TNR than “always-on” probes based on these strategies; on the other hand, the selectivity of such probes to disease is relatively limited [55, 56]. Additionally, the quantum yield, photostability, and biocompatibility of activable fluorescent probes continue to pose a main challenge [55, 56]. Compared to the activable fluorescent probes, the “always-on” D-A-D type NIR-II fluorophores exhibit well-defined structures, minimal biotoxicity, good pharmacokinetics, and broader disease selectivity [57–59]. The NIR-II emission window enables the “always-on” D-A-D probes to overcome the interference of autofluorescence [60, 61]. Moreover, the PEG structure endows a prolonged circulation time that the “always-on” D-A-D probes can obtain higher TNR based on excellent tumor retention [62]. Therefore, exploring clinically relevant NIR-II probes may lead to the next generation of fluorescent bioimaging tracers for FGS.

**Scheme 1:**
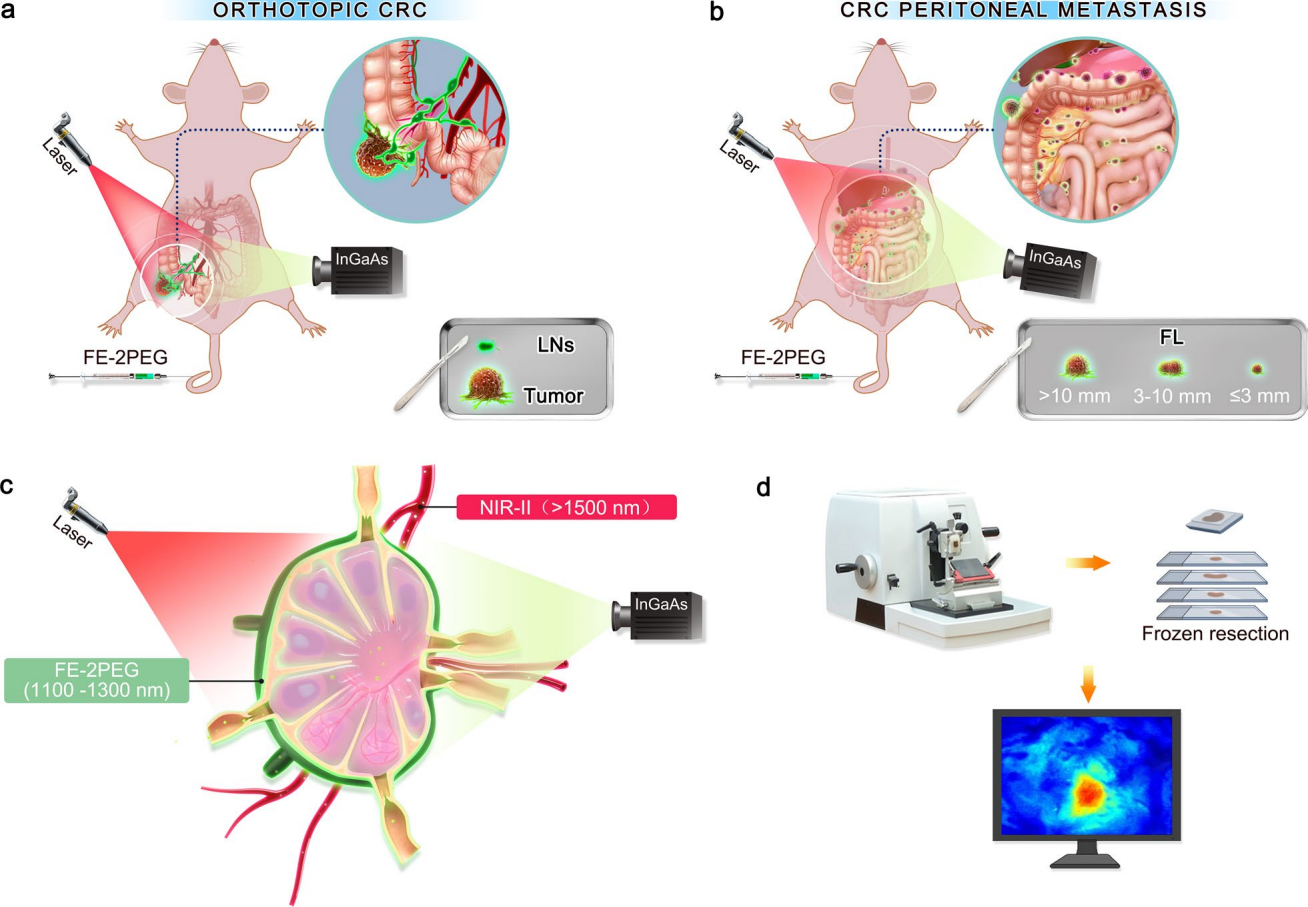
Schematic illustrating FE-2PEG for NIR-II FGS for CRC. **a** NIR-II FGS with D-A-D dye FE-2PEG of orthotopic CRC with lymphatic metastasis. LNs, lymph nodes. **b** NIR-II imaging navigation with FE-2PEG was used in FGS of PM. Supplementary biopsies with the naked eyes were carried out for diagnostic experiments. The nodules that FGS resected were classified into three subgroups according to the diameters for further analysis of the diagnostic experiment. FL, fluorescence. **c** Dual-NIR-II color probes were designed with D-A-D dye FE-2PEG at the NIR-IIa window and PbS@CdS QDs at the NIR-IIb window for simultaneously visualizing the mesenteric LNs and surrounding blood vessels. **d** NIR-II microscopic fluorescence images for intraoperative diagnosis

To the best of our knowledge, the application of NIR-II bioimaging in CRC intraoperative navigation has rarely been reported. In this study, we have rationally and innovatively constructed an efficiently dual-color platform consisting of organic D-A-D probe FE-2PEG (NIR-IIa) and PbS@CdS QDs (NIR-IIb) (Scheme 1). Compared with the clinical-using ICG and protein-based BSA@IR-780, FE-2PEG can be efficiently delivered into tumor sites and metastatic nodules via the vasculature system, utilizing the potent EPR effect. The superior photostability of FE-2PEG can efficiently resist photobleaching to obtain a prolonged surgical time window. In addition, the excellent biocompatibility endowed FE-2PEG with great potential for clinical translation. Overall, FE-2PEG has successfully enabled precise identification and real-time surgical navigation for orthotopic tumors, metastatic LNs, and peritoneal metastases of CRC.

## Results and discussion

### Characterization of FE-2PEG for NIR-II imaging-guided surgery

**Fig. 1:**
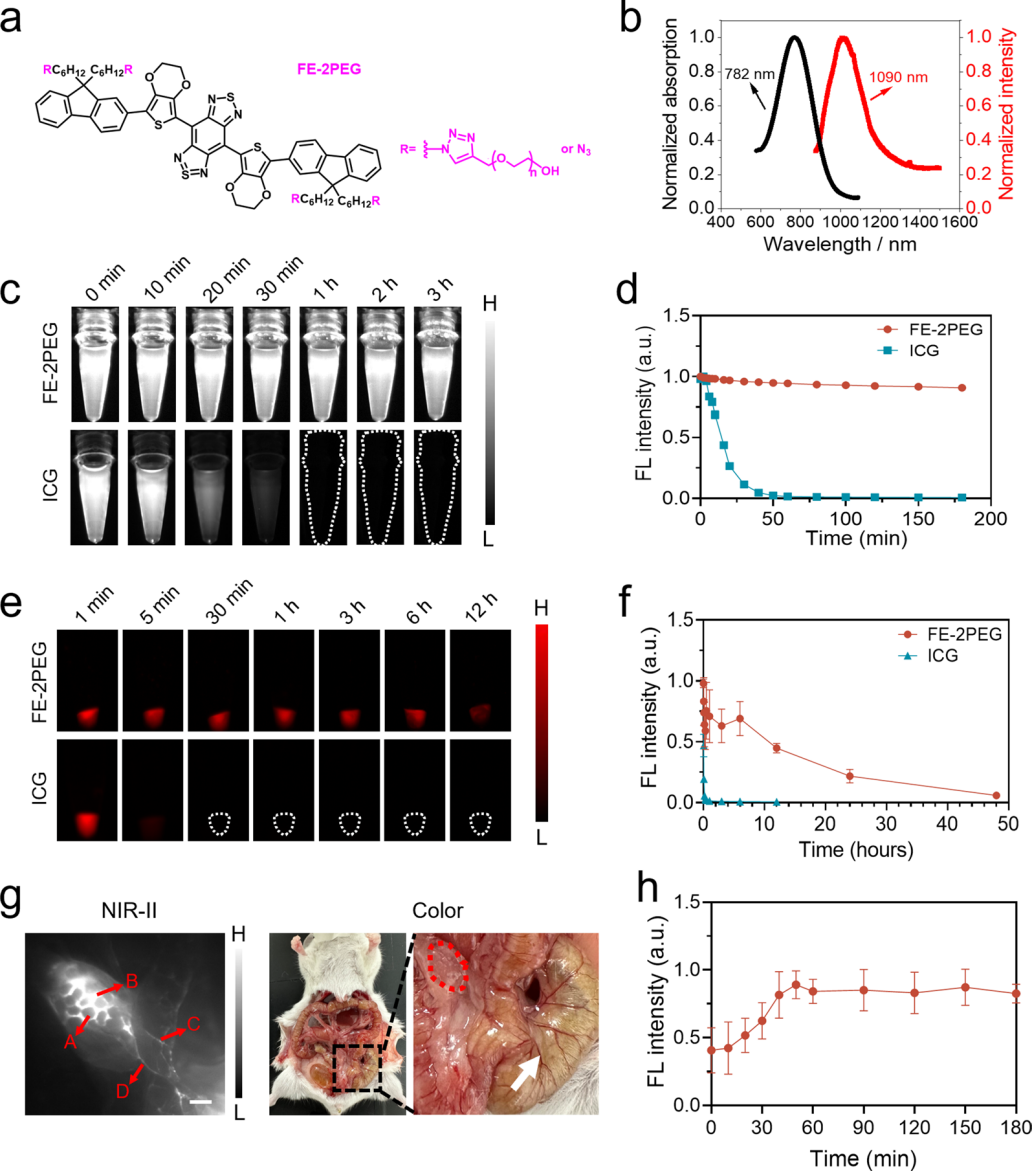
Characterization and in vivo NIR-II imaging of FE-2PEG. **a** Molecular structure of FE-2PEG. **b** Absorption and emission spectra of FE-2PEG. **c** The NIR-II fluorescence images of FE-2PEG and ICG under continuous irradiation of 808 nm laser at different time points. 65 mW/cm^2^; Over 1100 nm collection. **d** The normalized fluorescent intensity analysis of (**c**). **e** The NIR-II fluorescence images of the mice’s blood post-injection of FE-2PEG and ICG at different time points. (f) The qualitative and normalized fluorescent intensity analysis of (**e**) (n = 3, data are shown as mean ± SD). **g** Representative NIR-II imaging of mesenteric LNs post-injection of FE-2PEG at the cecum; The outline profiles of the lymphoid follicles (**A**) were delineated, and the lymphoid sinus (**B**), the afferent (**C**) and efferent lymphatic vessels (**D**) were illuminated. Scale bar: 1 mm. In the photograph of exposed mice, the white arrow shows the local injection site, and the red dotted line shows the mesenteric lymph node. **h** The normalized fluorescent intensity curve of mesenteric LNs (n = 5, data are shown as mean ± SD)

We selected a D-A-D probe FE-2PEG as it was proven with high and extremely stable NIR-II brightness, without any photobleaching concern. The molecular structure of FE-2PEG is shown in Fig. 1a. In PBS solution, the absorption spectrum of FE-2PEG exhibited a peak at 782 nm, while the fluorescence spectrum showed a peak at 1090 nm (Fig. 1b). The molecular structure of ICG and BSA@IR-780 are illustrated in Additional file 1: Fig. S1a, b. The optical characterization of ICG in PBS showed a strong absorption peak at 779 nm and a fluorescence peak at 909 nm, respectively (Additional file 1: Fig. S1c). BSA@IR-780 exhibited an absorption peak at 787 nm and a fluorescence peak at 904 nm, respectively (Additional file 1: Fig. S1d). We further constructed a NIR-II living imaging platform to measure the imaging capacity of FE-2PEG, as shown in Additional file 1: Fig. S2. To evaluate photostability, we subjected FE-2PEG and ICG to continuous irradiation using an 808 nm laser. The results showed that ICG was rapidly photobleached under laser irradiation, whereas the fluorescence signal of FE-2PEG maintained bright even after 3 h of continuous irradiation (Fig. 1c, d). To evaluate the photostability in vivo, popliteal and sacral LNs were irradiated continuously after being migrated by the FE-2PEG, ICG, and BSA@IR-780 from the foot pads, respectively. The results showed that the fluorescence intensity of the FE-2PEG only decreased by approximately 21% after two hours of irradiation. In contrast, the ICG and BSA@IR-780 decreased rapidly after 30 min of irradiation (Additional file 1: Figs. S3, S4). These results indicate that the FE-2PEG is remarkably stable, enabling long-term intraoperative imaging. We next evaluated the circulatory ability of FE-2PEG, ICG, and BSA@IR-780 by comparing the fluorescence intensity of blood at different time points after intravenous injection via the tail vein. The results revealed that the FE-2PEG retained 21.7% of its fluorescence intensity at 24 h post-injection, whereas ICG rapidly decreased to approximately zero within 10 min post-injection. This suggests that the FE-2PEG has a significantly prolonged blood circulation time compared to ICG, attributed to the PEG molecular structure (Fig. 1e, f). BSA@IR-780 also has excellent blood circulation ability, owing to the coating effect of albumin (Additional file 1: Fig. S5).

### High-contrast imaging of LNs and blood vessels by FE-2PEG

Based on the excellent photostability, the FE-2PEG was locally injected into the sub-serous layer of the cecum to visualize the mesenteric lymph node. Intriguingly, micro-structures of the mesenteric lymph node, including lymphoid follicles, lymphoid sinus, afferent and efferent lymphatic vessels, were clearly mapped (Fig. 1g). The fluorescent intensity curve of mesenteric LNs showed that the fluorescence peaked quickly at 50 min and steadily retained the observation window for up to 3 h (Fig. 1h, Additional file 1: Fig. S6). Moreover, the micro-structure of mesenteric LNs can be more clearly outlined by additional intermittent massages with the fluorescent signal increased continuously (Additional file 1: Fig. S7). These results imply that the FE-2PEG is an ideal contrast agent for imaging the lymphatic system. Subsequently, the imaging capability in the blood vessels was also evaluated, and the blood vessels remained visible for up to 4 h without significant reduction. In comparison, ICG has a short lifespan of approximately 30 min before it begins to decay (Additional file 1: Fig. S8). The FE-2PEG achieved a vascular SBR of 1.95 at 2 h, while ICG was unable to distinguish vessels from surrounding tissues (Additional file 1: Fig. S8). The prolonged circulatory time of FE-2PEG resulted in high-quality images of lymphatic vessels. Cerebral angiography is an excellent way to assess the imaging depth capability of probes. Thus, we performed NIR-II imaging of the cerebral blood vessels in mice under different long-pass filters (900, 1000, 1100, 1200, and 1300 nm). Gaussian fitting analysis showed that the full-width half maxima values of blood vessels decreased gradually as the NIR imaging windows shifted to longer wavelength (Additional file 1: Fig. S9). This led to a notable improvement in the clarity of the blood vessel contour and a significant enhancement of the imaging quality. The imaging capability of hind limb vessels of mice in vivo yielded similar results to cerebral angiography (Additional file 1: Fig. S10).

### NIR-II fluorescence imaging and surgical navigation of subcutaneous tumor of CRC

**Fig. 2:**
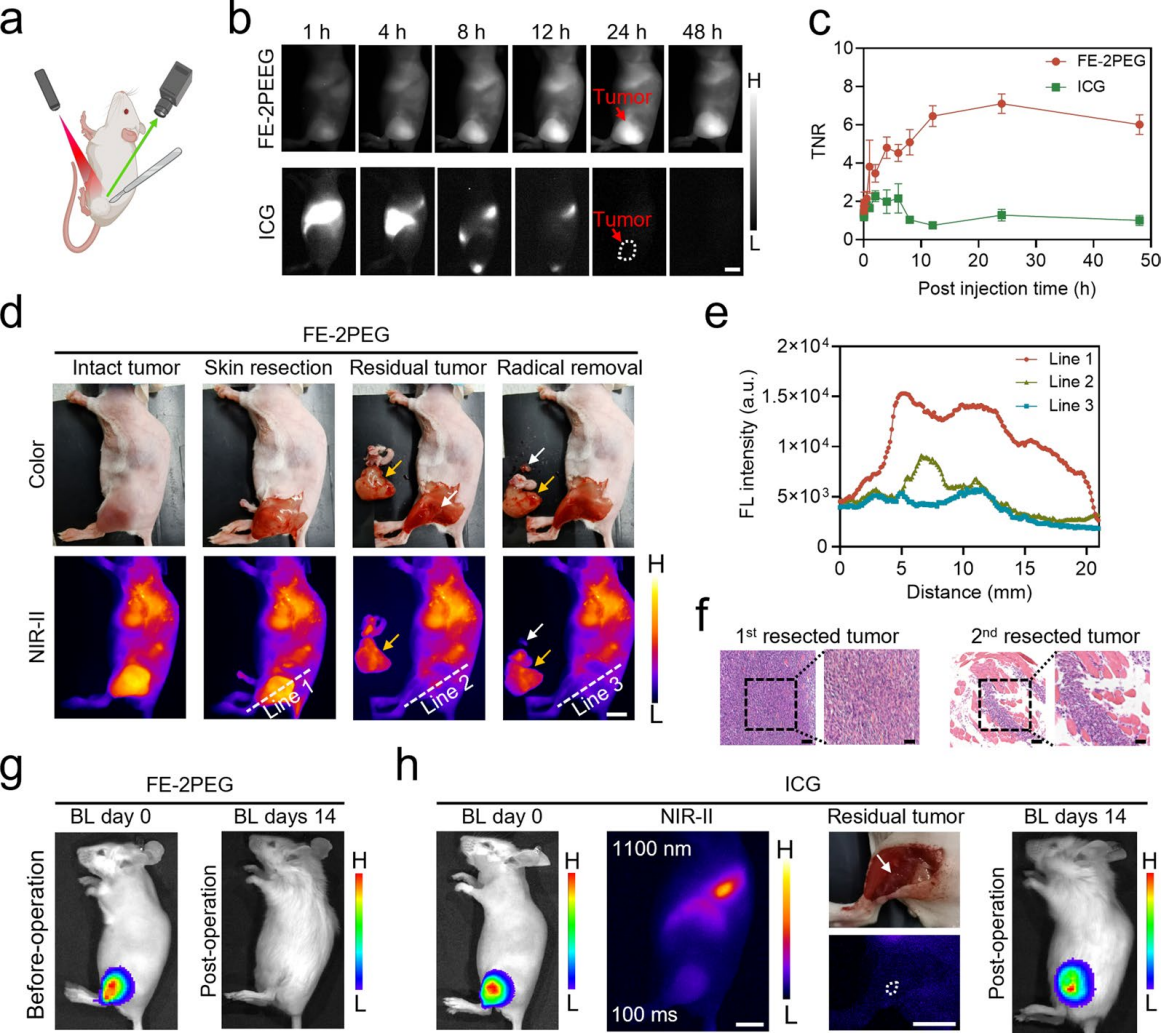
NIR-II in vivo FGS in subcutaneous tumors of CRC. **a** Schematic diagram of real-time surgical navigation of subcutaneous tumors of CRC. **b** NIR-II fluorescence images of CRC tumor-bearing mice at different time points post-injected of FE-2PEG and ICG via the tail vein. Scale bar: 1 cm. **c** The TNR curves at different time points of (**b**) (n = 3, data are shown as mean ± SD). **d** NIR-II FGS of CT-26-Luc tumors post-injection of FE-2PEG via the tail vein at 24 h. Over 1100 nm collection. Scale bar: 1 cm. **e** The profiles of the tumor with skin resection (line 1), the residual tumor (line 2), and after the radical removal (line 3) of (**d**). **f** H&E staining of the primary (yellow arrow) and residual tumors (white arrow). Scale bar: 100 μm (left); Scale bar: 50 μm (right). **g** BLI of CT-26-Luc tumors before (left) and after the operation with the guidance of FE-2PEG (14 days) (right). **h** The NIR-II images of CT-26-Luc tumors after tail vein injection of ICG at 24 h; the white light and NIR-II imaging of the residual tumor; BLI of CT-26-Luc tumors before (left) and after the operation with the guidance of ICG (14 days) (right). Scale bar: 1 cm

Next, we conducted further investigations into the tumor imaging capabilities of FE-2PEG and its potential from the real-time surgical navigation in the subcutaneous model of CRC (Fig. 2a). We respectively injected FE-2PEG and ICG into subcutaneous tumor-bearing mice via the tail vein. NIR-II photography was then performed at different time points to evaluate the tumor-targeting ability (Fig. 2b, c). The results revealed that FE-2PEG was highly effective in targeting tumors, with approximately a maximum TNR of 7.11 in vivo at 24 h. In contrast, ICG failed to illuminate the tumor (Fig. 2b, c). We also selected BSA@IR-780 as an alternative long-blood circulation probe and evaluated its enrichment ability in subcutaneous tumors. The results showed that BSA@IR-780 did not produce satisfactory TNR (Additional file 1: Fig. S11). To further evaluate the tumor-targeting ability of FE-2PEG, we anatomized tumor-bearing mice at 12 h, 24 h, and 48 h post-injection. The NIR-II imaging of organs (heart, liver, spleen, lung, kidney, normal intestine), tissues (skin and muscle), and tumors revealed that the probe predominantly accumulated in the tumor and liver (Additional file 1: Fig. S12).

Motivated by these findings, we performed real-time surgical navigation of subcutaneous CRC tumors using NIR-II fluorescence imaging guidance at 24 h. As shown in Fig. 2d, the tumor margins were clearly distinguished in both intact and incised skin situations. Leveraging the exceptional NIR-II imaging capability of the probe and the enhanced EPR effect, the residual tumor in millimeters was identified and completely resected by secondary surgery (Fig. 2d). Plot profiles of the tumor with skin incision (line 1), the residual tumor (line 2), and complete excision of the residual tumor (line 3) showed that there was still a significant peak in the signal corresponding to the presence of the residual tumor (line 2). The fluorescence intensity decreased significantly after the complete secondary surgery (line 3) (Fig. 2d, e). Hematoxylin-eosin (H&E) staining confirmed the resected tumor and the successful supplementary resection of the residual tumor (Fig. 2f). Compared to the bioluminescence imaging (BLI) of the tumor-bearing mice before surgery with 2 weeks after surgery, we found that the tumor was radically removed with no recurrence (Fig. 2g). Despite the exposure time being increased up to 100 ms, the poor EPR effect of ICG still resulted in indistinct tumor margins at the 24-h time point (Fig. 2h). It should be pointed out that the prolonged exposure time may cause visual delay and impede real-time surgery procedures. In addition, the residual tumor in millimeters appeared blurry and could not be accurately resected under fluorescent guidance, resulting in tumor recurrence after 2 weeks (Fig. 2h).

### Dual-color imaging and surgical navigation of orthotopic CRC

**Fig. 3:**
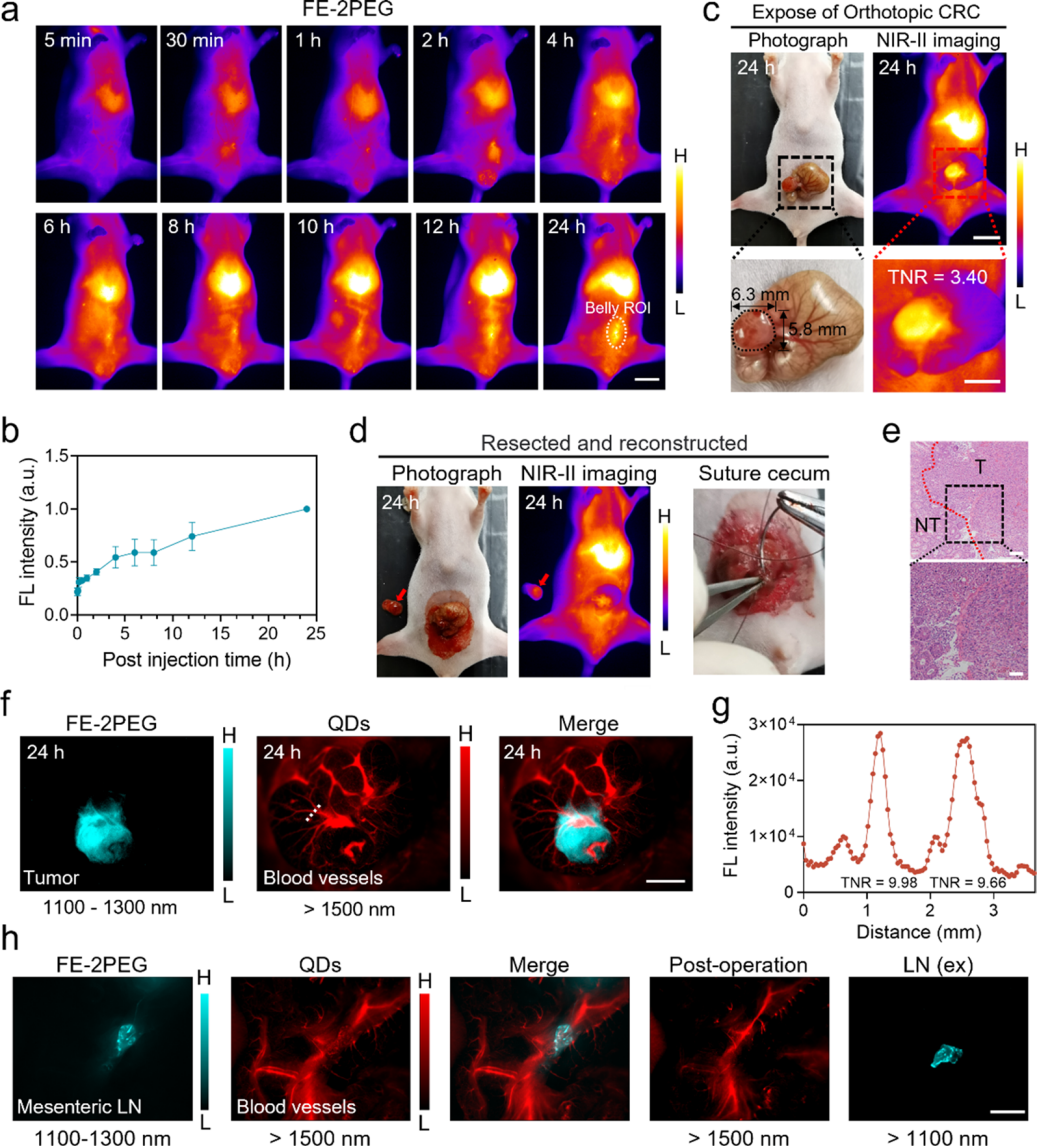
Dual-NIR-II color FGS of orthotopic CRC and mesenteric LNs. **a** NIR-II non-invasive in vivo imaging of orthotopic CRC mice post-injection of FE-2PEG via the tail vein at different time points. Scale bar: 1 cm. **b** Normalized FL intensity of the abdominal ROIs at different time points in (**a**) (n = 3, data are shown as mean ± SD). **c** White light and NIR-II imaging of exposed orthotopic CRC. The tumor size was 5.8 × 6.3 mm. The TNR was 3.40. Scale bar: 1 cm (top); Scale bar: 5 mm (down). **d** NIR-II FGS of the orthotopic tumors and reconstruction of the digestive tract. **e** H&E staining of the orthotopic tumor. The dotted line showed the tumor (T) and normal tissues (NT) boundary. Scale bar: 100 μm (up); Scale bar: 50 μm (down). **f** Dual-NIR-II color imaging of the orthotopic tumor and angiogenesis with FE-2PEG (1100 ~ 1300 nm) and QDs (> 1500 nm). Scale bar: 5 mm. **g** Profiles of the dotted line in (**f**). **h** Dual-NIR-II color imaging of the mesenteric lymph node and mesenteric blood vessels with FE-2PEG (1100 ~ 1300 nm) and QDs (> 1500 nm). Under the dual-NIR-II color navigation, the mesenteric lymph node was surgically excised, and the mesenteric blood vessels were protected. Scale bar: 5 mm

To test the suitability of FE-2PEG for imaging deep tumors, an orthotopic CRC model was established to estimate the effectiveness of NIR-II imaging and surgical navigation in deep tumors. The cecum of mice was exposed, and 2 × 10^6^ CT-26-Luc cells were injected into the subserous layer under the stereoscopic microscope (Additional file 1: Fig. S13). The growth of orthotopic CRC was monitored every 3 days using the IVIS Lumina III (PerkinElmer) system (Additional file 1: Fig. S14). The orthotopic deep-seated tumors were diagnosed non-invasively with FE-2PEG (300 µM, 300 µl) under NIR-II imaging (Fig. 3a, b). The fluorescence intensity of the abdominal region of interest (ROI) gradually enhanced and peaked at 24 h (Fig. 3a, b). The orthotopic tumor (5.8 × 6.3 mm) was exposed by incising the abdominal wall and illuminated with a TNR of 3.40 under NIR-II imaging (Fig. 3c). We performed precise resection of the tumors under real-time NIR-II fluorescence navigation, followed by tight suture of the cecum and closure of the abdominal wall (Fig. 3d). Post-operative mice showed a significant reduction in NIR-II signal in the belly ROI region compared to pre-operative mice (Additional file 1: Fig. S15). The H&E results demonstrated that the tumors were completely resected with negative margins (Fig. 3e).

To prevent accidental injuries and protect the blood supply, we utilized a dual-NIR-II color strategy to simultaneously image the surrounding vasculature with a high resolution during surgery. Ex vivo dual-NIR-II color testing of FE-2PEG, QDs, and mixture showed that the signal emitted by FE-2PEG is best collected in the 1100 ~ 1300 nm range, while QDs perform optimally over 1500 nm (Additional file 1: Fig. S16). The FE-2PEG illuminated the clear outline of orthotopic CRC within the 1100 ~ 1300 nm window (Fig. 3f). The QDs delineated the peripheral blood vessels around the tumor, achieving a TNR of approximately ten within the over 1500 nm window (Fig. 3f, g). Radical lymphadenectomy in colectomy may cause accidental bleeding due to the adjacent anatomy of mesenteric LNs and vessels (Fig. 3h). To address this, we locally injected FE-2PEG (1100 ~ 1300 nm) to illuminate the mesenteric lymph node, while QDs (> 1500 nm) were used to lighten the mesenteric blood vessels (Fig. 3h). Subsequently, we successfully removed the mesenteric lymph node and safeguarded the blood vessels from harm using dual-NIR-II guidance (Fig. 3h).

As a comparison, the surgical navigation capability of ICG and BSA@IR-780 was evaluated in the orthotopic CRC model (Additional file 1: Figs. S17a-b, S18a-b). When ICG was injected through the tail vein, there was no noticeable increase in NIR-II signal in the abdominal ROI of orthotopic CRC mice for up to 24 h (Additional file 1: Fig. S17c). Even with an increased exposure time of up to 100 ms, the exposed orthotopic tumor did not show significant fluorescence enhancement at 24 h (Additional file 1: Fig. S17d). The anatomy showed that the tumor’s fluorescent intensity is weaker than feces and organs (heart, liver, spleen, lung, kidney, et al.) (Additional file 1: Fig. S17e, f). The H&E staining confirmed the existence of tumor cells in the cecum (Additional file 1: Fig. S17g). Then, NIR-II imaging of BSA@IR-780 in the orthotopic tumor-bearing mice was intermittently monitored for 24 h (Additional file 1: Fig. S18c). However, quantitative analysis of the belly ROI did not show a significant increase in the signal that could aid in the non-invasive diagnosing of deep-seated lesions (Additional file 1: Fig. S18d). The orthotopic tumor was indistinguishable from the surrounding tissues in the NIR-II imaging at 24 h (Additional file 1: Fig. S18e). After the anatomy separation of the feces from the cecum, the ex vivo tumor only showed faint light, which was insufficient for imaging-guided surgery (Additional file 1: Fig. S18f). The profiles of three lines with arrows representing tumor in vivo (line 1), feces (line 2), and tumor ex vivo (line 3) showed that the fluorescence mainly originated from the feces rather than the orthotopic tumor (Additional file 1: Fig. S18g). Compared to the organs such as the heart, liver, spleen, lung, and kidney et al., the fluorescent intensity of orthotopic tumor was too weak to be recognized (Additional file 1: Fig. S18h). The H&E staining confirmed the existence of tumor cells in the cecum (Additional file 1: Fig. S18i).

### Identification and resection of orthotopic CRC with metastatic LNs

**Fig. 4:**
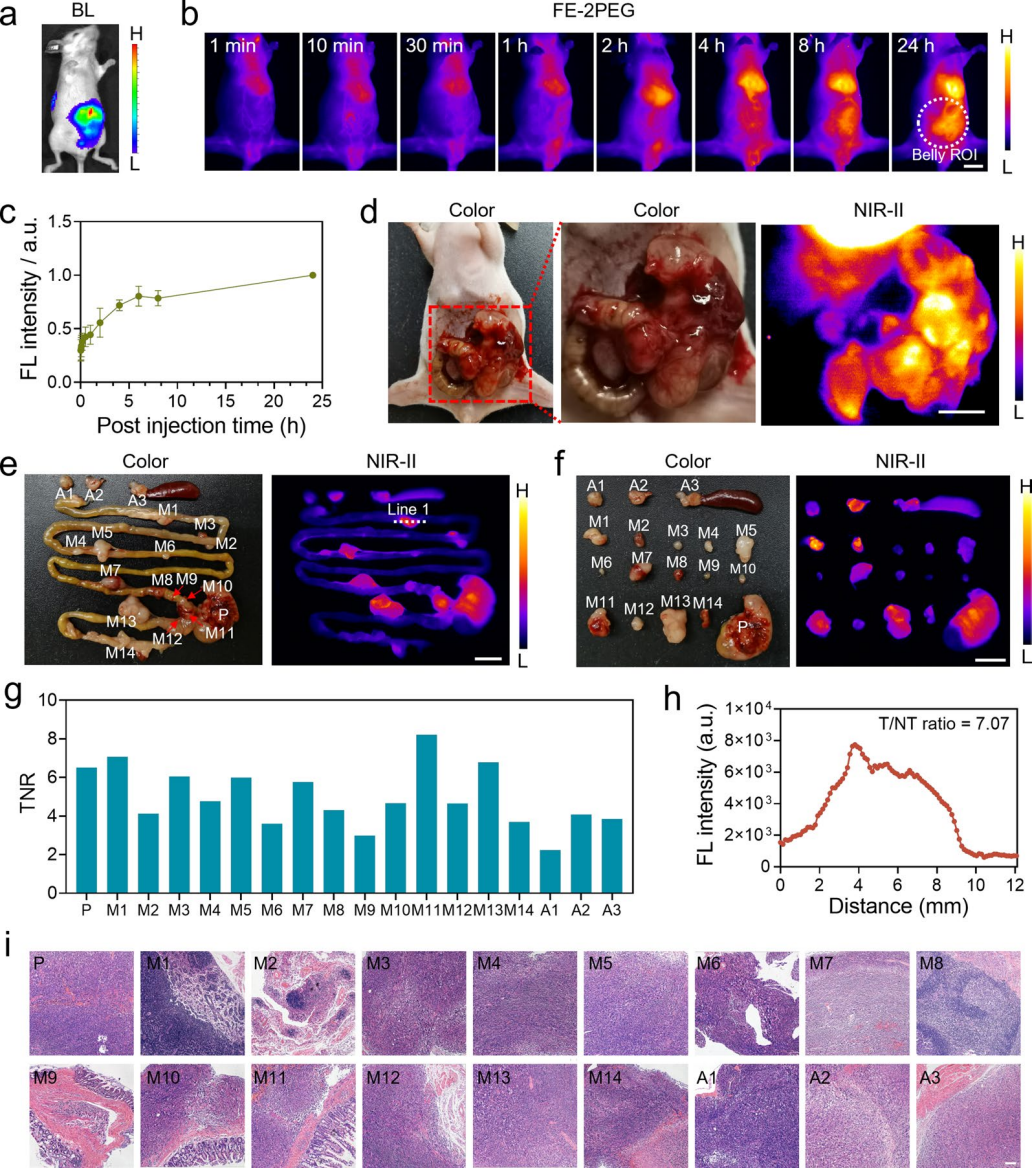
NIR-II imaging and FGS of orthotopic CRC with mesenteric lymphatic metastases. **a** BLI of orthotopic CRC with mesenteric lymphatic metastases. **b** The representative NIR-II in vivo imaging of orthotopic CRC with mesenteric lymphatic metastases at different time points post-injected with FE-2PEG via tail vein. Scale bar: 1 cm. **c** Quantitative analysis of fluorescence intensity of the abdominal ROIs at different time points in (**b**) (n = 3, data are shown as mean ± SD). **d** White light and NIR-II imaging of exposed tumors after laparotomy in vivo. Scale bar: 5 mm. **e** White light and NIR-II imaging of the orthotopic tumor (P), mesenteric lymphatic metastases (M1 ~ M14), and abdominal metastases (A1 ~ A3) ex vivo. Scale bar: 1 cm. **f** White light and NIR-II imaging of isolated orthotopic tumor (P), mesenteric lymphatic metastases (M1 ~ M14), and abdominal metastases (A1 ~ A3) ex vivo. Scale bar: 1 cm. **g** TNR of the primary lesion (P), mesenteric lymphatic metastases (M1 ~ M14), and abdominal metastases (A1 ~ A3). **h** Plot profiles of the representative mesenteric lymphatic metastasis (Line 1) in (**e**). **i** H&E staining of the primary tumor (P), mesenteric lymphatic metastases (M1 ~ M14), and abdominal metastases (A1 ~ A3). Scale bar: 100 μm

Lymphatic metastasis is one of the essential pathways in CRC that severely hinders patients’ prognosis. The orthotopic CRC model with lymphatic metastasis was successfully established by injecting a tumor cell suspension at the mesenteric side. BLI only showed a diffuse signal intensity change in the abdominal ROI with limited temporal-spatial resolution (Fig. 4a). The NIR-II imaging showed a more extensive and significant signal enhancement in the abdominal ROI 24 h after injection of FE-2PEG via the tail vein (Fig. 4b, c). In vivo imaging revealed significant probe accumulation in the primary tumor and multiple metastases (Fig. 4d). Through anatomical examination, the primary tumor (P), fourteen mesenteric metastatic LNs (M1-14) scattered throughout the digestive tract, two abdominal metastases below the diaphragm (A1-2), and one abdominal metastasis at the superior pole of the spleen (A3) were precisely recognized (Fig. 4e). After separating the primary tumor and metastases, we found that metastatic LNs were as small as 1.5 mm (M6, M9) (Fig. 4f). The TNR of the primary and all metastatic nodules exceeded 2.9 (Fig. 4g). Among these metastases, even the M6 and M9, with a tumor diameter of only 1.5 mm, had the TNR of 3.60 and 2.99, respectively (Fig. 4g). The profile of the representative M1 (TNR = 7.07) with a tumor diameter of 6.5 mm is shown in Fig. 4h. H&E staining confirmed the accurate diagnosis and complete resection of the primary tumor and metastatic nodules (Fig. 4i).

ICG and BSA@IR-780 were used in the model of primary tumors with metastatic LNs for comparison. However, both ICG and BSA@IR-780 exhibited lower TNR in the primary tumor and metastatic LNs, resulting in high interference and making it challenging to distinguish between tumors and tissues. Even with an increased exposure time of 200 ms, ICG was unable to provide imaging-guided diagnosis in vivo or ex vivo (Additional file 1: Fig. S19). Similarly, experiments on BSA@IR-780 failed to diagnose primary tumors and metastatic LNs in both in vivo or ex vivo NIR-II imaging (Additional file 1: Fig. S20).

### NIR-II microscopic imaging of primary tumor and metastatic nodules

**Fig. 5:**
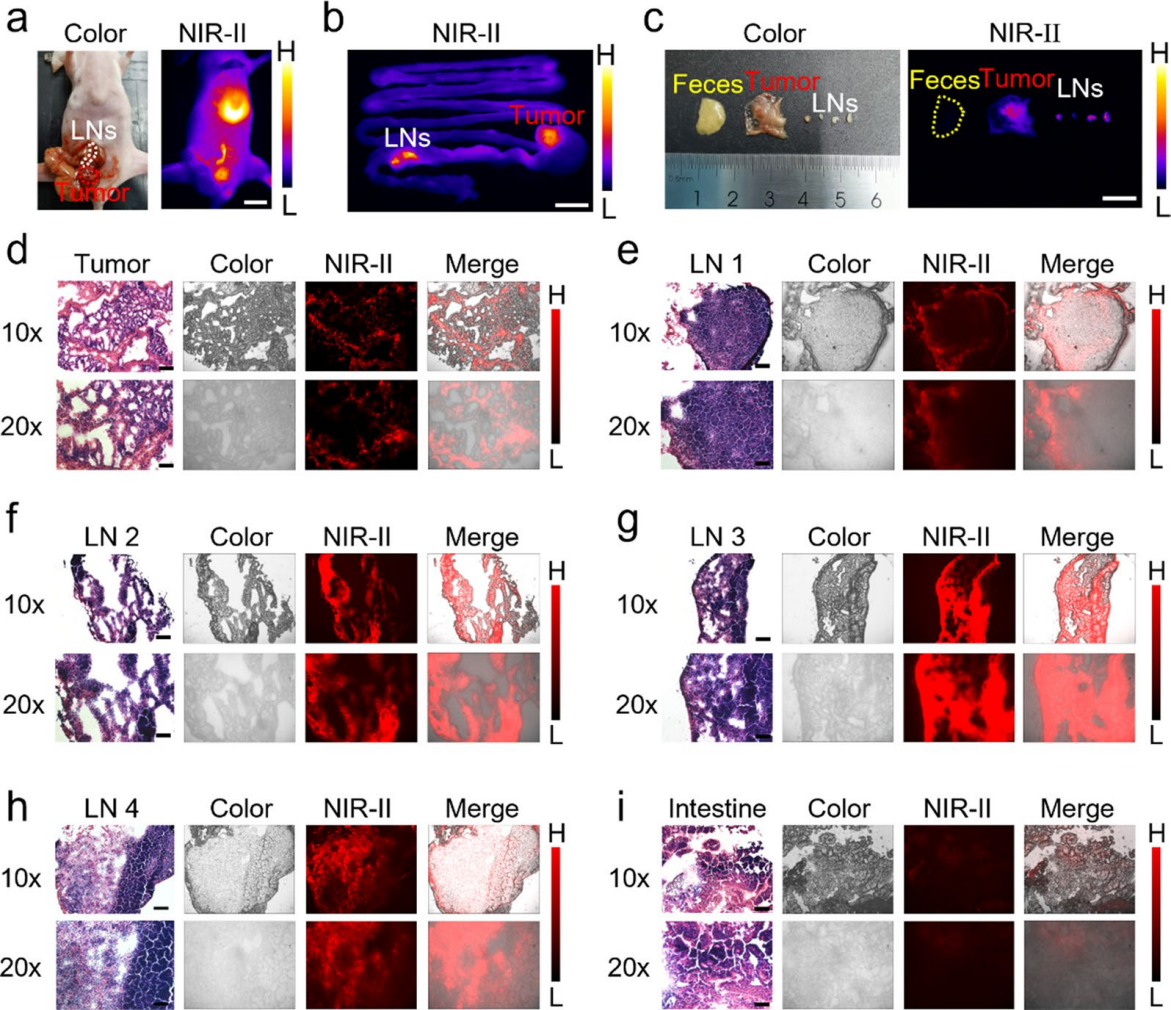
NIR-II microscopic imaging of orthotopic CRC with lymphatic metastases. **a** White light and NIR-II in vivo imaging of orthotopic CRC with lymphatic metastases post-injection of FE-2PEG via the tail vein at 24 h. Scale bar: 1 cm. **b** NIR-II imaging of orthotopic tumor and mesenteric lymphatic metastases ex vivo. Scale bar: 1 cm. **c** White light and NIR-II imaging of the isolated orthotopic tumor, LNs, and feces ex vivo. Scale bar: 1 cm. **d** The H&E staining, white light microscopic, NIR-II microscopic, and the merged imaging of the orthotopic tumor. Scale bar: 100 μm (10x); Scale bar: 50 μm (20x). **e** The H&E staining, white light microscopic, NIR-II microscopic, and the merged imaging of the lymph node 1 (LN 1). Scale bar: 100 μm (10x); Scale bar: 50 μm (20x). **f** The H&E staining, white light microscopic, NIR-II microscopic, and the merged imaging of the lymph node 2 (LN 2). Scale bar: 100 μm (10x); Scale bar: 50 μm (20x). **g** The H&E staining, white light microscopic, NIR-II microscopic, and the merged imaging of the lymph node 3 (LN 3). Scale bar: 100 μm (10x); Scale bar: 50 μm (20x). **h** The H&E staining, white light microscopic, NIR-II microscopic, and the merged imaging of the normal intestinal canal. Scale bar: 100 μm (10x); Scale bar: 50 μm (20x)

We further investigated the NIR-II microscopic imaging of FE-2PEG in primary CRC tumors with metastatic LNs. In vivo and ex vivo imaging of the primary tumor and metastatic LNs illuminated a sharp outline after injection of FE-2PEG at 24 h (Fig. 5a, b). Using NIR-II real-time navigation, the syncretic metastatic LNs were recognized and dissected into four isolated LNs (1 to 2 mm in diameter) (Fig. 5c). Frozen sections of the primary tumor, metastatic LNs, and normal intestine were examined using NIR-II microscopy. The NIR-II microscopic fluorescence significantly enhanced the signal in the primary tumor and four isolated metastatic LNs, while only a weak signal of the probe was observed in the normal intestine (Fig. 5d–i). H&E staining was performed to confirm the presence of tumor cells (Fig. 5d–i).

### Identification and precise resection of colorectal peritoneal metastatic nodes

**Fig. 6:**
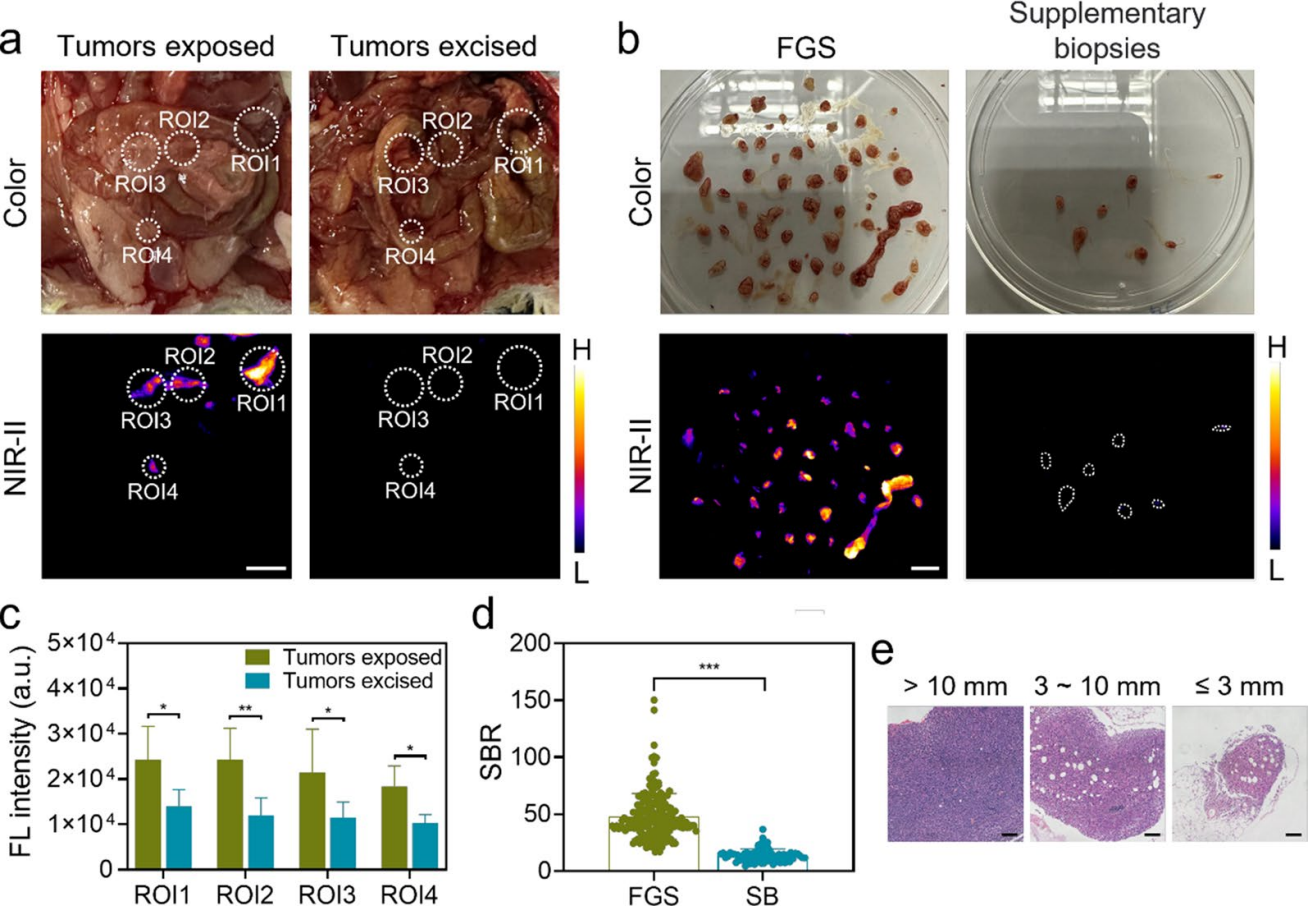
NIR-II imaging and surgical navigation of peritoneal metastases of CRC. **a** White light and NIR-II imaging of the tumors exposed and after tumors excised in vivo. Abdominal ROI regions 1, 2, 3, and 4 were quantitively analyzed. Scale bar: 5 mm. **b** White light and NIR-II imaging of lesions resected under fluorescent navigation and suspicious lesions with supplemental biopsies ex vivo. Scale bar: 5 mm. **c** Fluorescence intensity statistics of ROI regions (1, 2, 3, and 4) of tumors exposed and after tumors excised in (**a**) (data are shown as mean ± SD). **d**. SBR of lesions of FGS compared to suspicious lesions of supplemental biopsies (SB) in (**b**) (data were shown as mean ± SD). **e**. Representative H&E staining images of tumors with different diameters (> 10 mm, 3 ~ 10 mm, and ≤ 3 mm), Scale bar: 100 μm. *p < 0.05, **p < 0.01, ***p < 0.001

Micrometastases measuring less than 3 mm are difficult to distinguish and treat during cytoreductive surgery of CRC PM. Encouraged by the in vivo results, we aimed to explore the diagnostic and surgical navigational capacity of FE-2PEG in micrometastases. We established the PM model by intraperitoneal injection of CT-26-Luc cells and monitored the metastases by BLI (n = 8) (Additional file 1: Fig. S21). Non-invasive NIR-II live imaging showed a gradual fluorescent increase in abdominal ROI over time (Additional file 1: Fig. S22). Due to the excellent imaging depth of FE-2PEG, NIR-II fluorescence enhancement was detected even with an intact abdomen (Additional file 1: Fig. S22). During laparotomy, abdominal metastases could be clearly identified and precisely resected under real-time NIR-II fluorescence navigation (Fig. 6a). After surgery, there was a significant decrease in NIR-II fluorescent intensity in four abdominal ROI regions (Fig. 6a, c). The post-operative abdomen was examined and biopsied to identify any suspected missed lesions visible to the naked eyes. The fluorescence intensity of the NIR-II-guided resected lesions was significantly higher than that of supplemental biopsies (Fig. 6b, d). The resected nodules were classified based on tumor diameter (> 10 mm, > 3 mm and ≤ 10 mm, and ≤ 3 mm) and subjected to H&E staining as the gold standard for confirming the presence of tumor cells (Fig. 6e).

A total of 321 nodules were resected under fluorescent surgical navigation, with 310 lesions testing positive and 11 testing negative in the H&E examination. Additionally, a total of 89 nodules were resected by supplemental biopsies, with 18 testing positive and 71 testing negative in the H&E examination. With a 95% confidence interval, the sensitivity was calculated as 94.51% (92.03–96.99%), the specificity was calculated as 86.59% (79.05–94.12%), the positive predictive value (PPV) was calculated as 96.57% (94.57–98.57%), and the negative predictive value (NPV) was calculated as 79.78% (71.27–88.28%) (Table 1). Furthermore, we analyzed all nodules according to their diameter (> 10 mm, > 3 mm and ≤ 10 mm, and ≤ 3 mm). In the group with a tumor diameter > 10 mm, all 7 nodules were found to be positive in both NIR-II and H&E examinations, resulting in the sensitivity and PPV being 100%. In the group of nodules with a diameter between 3 and 10 mm, 85 NIR-II positive nodules had 83 positive and 2 negative results in the H&E examination, while 38 NIR-II negative nodules had 9 positive and 29 negative results. With a 95% confidence interval, the sensitivity was calculated as 90.21% (84.03–96.40%), the specificity was calculated as 93.55% (84.39–102.71%), the PPV was calculated as 97.65% (94.36–100.94%), and the NPV was calculated as 76.32% (62.15–90.48%). In the group with a tumor diameter ≤ 3 mm, 229 NIR-II positive nodules were found, with 220 testing positive and 9 testing negative in the H&E examination; 51 NIR-II negative nodules were found, with 9 testing positive and 42 testing negative in the H&E examination. With a 95% confidence interval, the sensitivity was calculated as 96.07% (93.53–98.61%), the specificity was calculated as 82.35% (71.52–93.18%), the PPV was calculated as 96.07% (93.53–98.61%), and the NPV was calculated as 82.35% (71.52–93.18%) (Table 2).

**Table 1:** NIR-II FGS diagnosis performance of FE-2PEG in the CT-26-Luc cell-derived PM model

		H&E		Sensitivity (95% CI)	Specificity (95% CI)	PPV (95% CI)	NPV (95% CI)
		Positive	Negative				
NIR-II	Positive	310	11	94.51 (92.03–96.99)	86.59 (79.05–94.12)	96.57 (94.57–98.57)	79.78 (71.27–88.28)
	Negative	18	71				

*CI* confidence limits, *PPV* positive predictive value, *NPV* negative predictive value; Florescent nodules and non-fluorescent biopsies of diaphragm, mesocolon, mesentery, omentum, and Douglas pouch

**Table 2:** NIR-II FGS diagnosis performance of FE-2PEG in different tumor diameters

	Nodule diameter		H&E		Sensitivity (95% CI)	Specificity (95% CI)	PPV (95% CI)	NPV (95% CI%)
			Positive	Negative				
NIR-II	> 10 mm	Positive	7	0	100	–	100	–
		Negative	0	0				
	3 mm < nodule ≤ 10 mm	Positive	83	2	90.21 (84.03–96.40)	93.55 (84.39–102.71)	97.65 (94.36–100.94)	76.32 (62.15–90.48)
		Negative	9	29				
	≤ 3 mm	Positive	220	9	96.07 (93.53–98.61)	82.35 (71.52–93.18)	96.07 (93.53–98.61)	82.35 (71.52–93.18)
		Negative	9	42				

*CI* confidence limits, *PPV* positive predictive value, *NPV* negative predictive value

#### Biosafety assessment of FE-2PEG

Excellent biocompatibility is a prerequisite for clinical application. The results of cellular activity using Cell Counting Kit-8 showed that both CT-26-Luc and L-929 cell lines maintained satisfactory cell viability after co-incubation with different concentrations of FE-2PEG for 12 and 24 h. The FE-2PEG maintained excellent biocompatibility even at the concentration of 20 µM (Additional file 1: Fig. S23). H&E staining of the major organs (heart, liver, spleen, lung, and kidney) harvested 30 days post-injection of FE-2PEG, ICG, and BSA@IR-780 confirmed the absence of any morphology abnormalities (Additional file 1: Fig. S24). Blood routine examination conducted over a period of 30 days for FE-2PEG, ICG, and BSA@IR-780 showed that the white blood cell, red blood cell, hemoglobin, hematokrit, mean corpuscular volume, mean corpuscular hemoglobin, mean corpuscular hemoglobin concentration, red blood cell distribution width, platelet, mean platelet volume, and platelet distribution width were all within the normal range (Additional file 1: Fig. S25a). Liver and kidney function tests over the same 30 days showed that alanine aminotransferase, aspartate aminotransferase, total bilirubin, albumin, alkaline phosphatase, γ-glutamyltranspeptidase, total bile acid, urea, creatinine, and uric acid were all within the normal range (Additional file 1: Fig. S25b). In addition, the change in body weight over a 2-week period showed no significant decrease post-injection of FE-2PEG, ICG, and BSA@IR-780 (Additional file 1: Fig. S25c). Based on the abovementioned evidence, FE-2PEG exhibited satisfactory biosafety with excellent potential for clinical application.

## Conclusion

In conclusion, we developed FE-2PEG for diagnosis and FGS of CRC. The FE-2PEG has prolonged circulation time, which enhances tumor imaging through the EPR effect. The efficient passive tumor targeting, stable tumor retention, and superior TNR enabled precise tumor resection under 808-nm laser irradiation. It also showed excellent biocompatibility and relatively higher-depth temporal-spatial resolution in NIR-II imaging. Identification and resection of regional metastatic LNs and detection of occult micrometastases in the peritoneum have implications for CRC diagnosis and prognosis. In this study, we successfully outlined 14 metastatic LNs with a minimum diameter of 1.5 mm and maximum TNR of 8.22 by virtue of effective tumor retention in one representative orthotopic CRC model. In the PM model, the fluorescent signal in four abdominal ROIs exhibited a significant decline after FGS (p < 0.05), which is conducive to intraoperative evaluation of whether micrometastases were thoroughly eliminated. Moreover, the ex vivo data confirmed that the SBR of nodules resected by FGS are much higher than those by supplementary biopsies (p < 0.05), which can improve the accuracy and reduce the invasiveness of surgery. Notably, even for micro-nodules smaller than 3 mm, the FE-2PEG can facilitate surgical delineation of mesenteric lymphatic and peritoneal micrometastases during the retention period, with a PPV value of up to 96.07%. Pathological staining of the removed nodules confirmed the successful delineation of tumor boundary from normal tissues by NIR-II FGS, facilitating complete tumor resection. Meanwhile, by combining NIR-IIb angiography, the dual-NIR-II imaging strategy enables the differentiation of peripheral blood vessels during the surgery of orthotopic CRC and mesenteric LNs, increasing surgical precision and minimizing damage to normal. More encouragingly, this NIR-II contrast agent exhibited satisfactory biocompatibility with no organ harm and long-term toxicity, thus presenting promising opportunities for future biomedical applications. Our study sheds light on the precise identification of surgical margins and micrometastases of CRC, thereby guiding and optimizing therapeutic procedures.

### Supplementary Information

**Additional file 1: Scheme S1.** Synthesis of IR-FE and IR-FE-N3. **Figure S1.** Characterization of ICG and BSA@IR-780. a. Schematic diagram of molecular structure of ICG. b. Schematic diagram of the molecular structure for BSA@IR-780. c. Absorption and emission spectra of ICG. d. Absorption and emission spectra of BSA@IR780. **Figure S2.** The scheme of the NIR-II imaging and operating platform. Created with BioRender.com. **Figure S3.** Photostability of FE-2PEG and ICG in popliteal and sacral lymph nodes. a. Continuously irradiation of popliteal and sacral lymph nodes under 808 nm laser. The left hind limb was injected with FE-2PEG (600 μM, 25 μL), and the right hind limb was injected with ICG (100 μM, 25 μL) in the footpad. > 1100 nm. 65 mW/cm^2^. Scale bar: 1 cm. b. The normalized fluorescence intensity curve of (a) (n = 3, data were shown as means ± SD). **Figure S4.** Photostability of BSA@IR-780 in popliteal and sacral lymph nodes in vivo. a. Continuously irradiation of popliteal and sacral lymph nodes under 808 nm laser. The bilateral hind limbs were injected with BSA@IR-780 (300 μM, 25 μL) in the footpad. > 1100 nm. 65 mW/cm^2^. Scale bar: 1 cm. b. The normalized fluorescence intensity curve of (a) (n = 3, data were shown as means ± SD). **Figure S5.** Evaluation of blood circulation time of BSA@IR-780. a. NIR-II imaging of blood ex vivo post-injected of BSA@IR-780 (150 μM, 200 μL) via tail vein at different time points. b. The normalized quantitative curve of fluorescence intensity of blood at different time points (n = 3, data were shown as means ± SD). **Figure S6.** Representative NIR-II images of mesenteric LNs at different time points. The FE-2PEG was injected into the subserous layer of the cecum (300 μM, 20 μL). The injection site was massaged for 3 min to facilitate the flow of the probe in the lymphatic fluid. Over 1100 nm collection; 65 mW/cm^2^; Scale bar: 1 mm. **Figure S7.** The NIR-II imaging of mesenteric LNs at different time points. a. Representative NIR-II images of mesenteric LNs. The probe FE-2PEG was injected into the subserous layer of the cecum (300 μM, 20 μL). The injection site was massaged for 3 min at the start, and then intermittently massaged for 30 seconds before each capture. b. The normalized fluorescent intensity curve of mesenteric LNs (n=5, data were shown as mean ± SD). Over 1100 nm collection; 65 mW/cm^2^; Scale bar: 1 mm. **Figure S8.** NIR-II imaging of FE-2PEG and ICG in blood vessels of the hindlimb. a. Representative NIR-II imaging of FE-2PEG and ICG in blood vessels of the hindlimb at different time points. Scale bar: 5 mm. b. Normalized FL intensity in vessels of FE-2PEG and ICG. c. Plot profiles of lines on blood vessels of hindlimb at 2 hours (n = 3, data were shown as means ± SD). **Figure S9.** The wavelength-dependence NIR-II imaging of FE-2PEG in cerebral vessels. a. Representative images of the cerebral vessels in NIR-II (900 nm, 1000 nm, 1100 nm, 1200 nm, and 1300 nm) windows. Scale bar: 5 mm. b. Fluorescence cross-sectional intensity distribution of cerebral vessels in different NIR-II windows (red line in (a)) (n = 3). The full-width half maxima (FWHM) are 0.81124, 0.80007, 0.7731, 0.71646, and 0.69999 for 900 nm, 1000 nm, 1100 nm, 1200 nm, and 1300 nm, respectively. **Figure S10.** The wavelength-dependence NIR-II imaging of FE-2PEG in blood vessels of the hindlimb. a. Representative images of blood vessels in the hindlimb in NIR-II (900 nm, 1000 nm, 1100 nm, 1200 nm, and 1300 nm) windows. Scale bar: 5 mm. b. Fluorescence cross-sectional intensity distribution of blood vessels in hindlimb in different NIR-II windows (red line in (a)) (n = 3). The full-width half maxima (FWHM) are 0.86926, 0.77146, 0.74858, 0.65666, and 0.55798 for 900 nm, 1000 nm, 1100 nm, 1200 nm, and 1300 nm, respectively.** Figure S11.** NIR-II in vivo imaging of BSA@IR-780 in the subcutaneous tumor. a. Representative NIR-II in vivo images of BSA@IR-780 (150 μM, 200 μL) in subcutaneous tumor at different time points. Lateral position. Tail vein injection. Scale bar: 1 cm. b. The T/NT ratio of subcutaneous tumors at different time points in vivo. **Figure S12.** Evaluation of the biodistribution of FE-2PEG. a. White light and NIR-II imaging of the tumor, organs (heart, liver, spleen, lung, kidney, intestine), and tissues (skin, muscle) at 12h. Scale bar: 1 cm. b. White light and NIR-II imaging of the tumor, organs, and tissues at 24h. Scale bar: 1 cm. c. White light and NIR-II imaging of the tumor, organs, and tissues at 48h. Scale bar: 1 cm. d. Quantitative analysis of fluorescence intensity signals of tumors, organs, and tissues at different time points (n = 3, data were shown as means ± SD).** Figure S13.** The establishment of the orthotopic mouse model of CRC. a. The preparation of surgical instruments and materials. Materials for surgery include cotton (A), cotton swabs (B), gauze (C), and absorbable sutures (D). Instruments for surgery include bent scissor (E), needle holder (F), straight tweezer (G), bent tweezer (H), and micro-syringe (I). b. The surgical operating platform includes the stereomicroscope (A), operating table (B), monitor (C), and LED lamp (D). c. The cecum was exposed through an abdominal incision. The dotted line shows the cecum. The gauze was used to protect the cecum. d. The CT-26-Luc cells were injected into the subserous membrane of the cecum with the micro-syringe. The red arrow shows tumor cells. e. The incisions in the abdomen were sutured layer by layer with absorbable sutures. The dotted line shows the abdominal incision after the suture. **Figure S14.** BLI monitoring of the orthotopic CRC model formation. **Figure S15.** The NIR-II imaging of post-operative orthotopic CRC mice. a. The photograph of post-operative orthotopic CRC mice. b. Representative NIR-II imaging of post-operative orthotopic CRC mice post-injected with FE-2PEG via the tail vein at 24 hours. Scale bar: 1 cm. **Figure S16.** Dual-NIR-II color testing of FE-2PEG, QDs, and mixture in vitro. a. The photograph of the FE-2PEG, QDs, and mixture solutions. b. The NIR-II fluorescent signals were collected under > 1100, 1100 ~ 1300, and > 1500 nm windows, respectively. **Figure S17.** NIR-II imaging of ICG in orthotopic CRC in vivo. a. The schematic diagram showed that the orthotopic CRC mouse was injected with ICG (150 μM, 200 μL) via the tail vein. b. BLI showed the successful establishment of the orthotopic CRC model. c. Representative images of orthotopic CRC post-injected ICG at different time points in vivo. Scale bar: 1 cm. d. White light and NIR-II imaging of exposed orthotopic CRC in vivo. > 1100 nm. Exposure time = 100 ms. Scale bar: 5 mm. e. White light and NIR-II imaging of the organs and tumor ex vivo > 1100 nm, exposure time = 100 ms. Scale bar: 1 cm. f. The fluorescence intensity ratio of tumor to feces ex vivo. (n = 3, data were shown as means ± SD). g. H&E staining of the orthotopic tumor. Scale bar: 100 μm (10x); Scale bar: 50 μm (20x). **Figure S18.** NIR-II imaging of BSA@IR-780 in orthotopic CRC. a. The schematic diagram showed that the orthotopic CRC mouse was injected with BSA@IR-780 (150 μM, 200 μL) via the tail vein. b. BLI showed the successful establishment of the orthotopic CRC model. c. Representative NIR-II images of BSA@IR-780 in orthotopic CRC at different time points with a supine position. Scale bar: 1 cm. d. Quantitative intensity signal analysis of abdominal ROI. e. White light and NIR-II imaging of the exposed orthotopic tumor in vivo. The tumor size is 4.2 mm in length and 4.1 mm in width. Scale bar: 5 mm. f. White light and NIR-II imaging of the isolated tumor and feces ex vivo. Scale bar: 5 mm. g. Plot profiles quantitative analysis of the tumor in vivo (line 1), the isolated feces (line 2), and the tumor ex vivo (line 3). h. White light and NIR-II imaging of isolated organs, feces, and the tumor ex vivo. Scale bar: 1 cm. i. H&E staining of the orthotopic tumor. Scale bar: 100 μm (10x); Scale bar: 50 μm (20x). **Figure S19.** NIR-II imaging of ICG in orthotopic CRC with lymphatic metastases. a. White light and NIR-II imaging of orthotopic tumor and metastatic LNs before and after laparotomy in vivo. Scale bar: 1 cm. b. White light and NIR-II imaging of intestinal canal, orthotopic tumor, and metastatic LNs ex vivo. Scale bar: 1 cm. c. T/NT ratio of the orthotopic tumor and metastatic LNs in vivo and ex vivo (n = 3, data were shown as means ± SD). **Figure S20.** NIR-II imaging of BSA@IR-780 of orthotopic CRC with lymphatic metastases. a. White light and NIR-II imaging of primary tumor and metastatic lymph nodes displayed before and after laparotomy in vivo. Scale bar: 1 cm. b. White light and NIR-II imaging of intestinal canal, orthotopic tumor, metastatic LNs, and feces ex vivo. Scale bar: 1 cm. c. T/NT ratio of the orthotopic tumor and metastatic LNs in vivo and ex vivo. **Figure S21.** BLI of the establishment of CRC peritoneal metastasis model. **Figure S22.** NIR-II in vivo imaging of FE-2PEG in CRC peritoneal metastasis model. a. Representative NIR-II images of FE-2PEG in peritoneal metastasis mice at different time points. Supine position,tail vein injection, 300 μM, 300 μL. Scale bar: 1 cm. b. Quantitative signal intensity analysis of abdominal ROI. **Figure S23.** Biological safety of FE-2PEG. a. CCK-8 viabilities of CT-26-Luc cells after incubated with various concentrations of FE-2PEG for 12 h and 24 h; b. Viabilities of L-929 cells after incubation with various concentrations of FE-2PEG for 12 h and 24 h. (n = 6, data were shown as means ± SD). **Figure S24.** Biological safety of FE-2PEG, ICG, and BSA@IR-780. a. H&E staining of organs (Heart, liver, spleen, lung, and kidney) from mice after intravenous injection of FE-2PEG (300 μM, 300 μL), ICG (150 μM, 200 μL), and BSA@IR-780 (150 μM, 200 μL) in 30 days (n = 3). Scale bar: 100 μm. **Figure S25.** Biological safety of FE-2PEG, ICG, and BSA@IR780. a. Blood routine examination in 30 days post-injected of FE-2PEG (300 μM, 300 μL), ICG (200 μM, 150 μL), and BSA@IR-780 (200 μM, 150 μL) via tail vein. (n = 3, data were shown as means ± SD). b. Liver and renal function examination in 30 days post injection of FE-2PEG (300 μM, 300 μL), ICG (200 μM, 150 μL), and BSA@IR-780 (200 μM, 150 μL) via tail vein. (n = 3, data were shown as means ± SD). c. Body weight change in two weeks post-injected of FE-2PEG (300 μM, 300 μL), ICG (200 μM, 150 μL), and BSA@IR-780 (200 μM, 150 μL) via tail vein. (n = 3, data were shown as means ± SD).

## Data Availability

The datasets used and/or analyzed during the current study are available from the corresponding author on reasonable request.

## Abbreviations

CRC: Colorectal cancer
PSM: Positive surgical margin
LNs: Lymph nodes
PM: Peritoneal metastasis
FGS: Fluorescence-guided surgery
NIR-I: The first near-infrared window
NIR-II: The second near-infrared window
TNR: Tumor-to-normal tissue ratio
ICG: Indocyanine green
BSA: Bovine serum albumin
QDs: Quantum dots
D-A-D: Donor-pi-acceptor-pi-donor
EPR: Enhanced permeability and retention
PEG: Poly (ethylene glycol)
SBR: Signal-to-background ratio
H&E: Hematoxylin-eosin
BLI: Bioluminescence imaging
ROI: Region of interest
FL: Fluorescence
