# Supplementary material for: High-precision detection and navigation surgery of colorectal cancer micrometastases
Source: J Nanobiotechnology. 2023 Nov 2;21:403. doi: 10.1186/s12951-023-02171-z (PMC10621104; doi:10.1186/s12951-023-02171-z)
Supplement: Supplementary file 1 — Additional file 1: Scheme S1. Synthesis of IR-FE and IR-FE-N3. Figure S1. Characterization of ICG and BSA@IR-780. a. Schematic diagram of molecular structure of ICG. b. Schematic diagram of the molecular structure for BSA@IR-780. c. Absorption and emission spectra of ICG. d. Absorption and emission spectra of BSA@IR780. Figure S2. The scheme of the NIR-II imaging and operating platform. Created with BioRender.com. Figure S3. Photostability of FE-2PEG and ICG in popliteal and sacral lymph nodes. a. Continuously irradiation of popliteal and sacral lymph nodes under 808 nm laser. The left hind limb was injected with FE-2PEG (600 μM, 25 μL), and the right hind limb was injected with ICG (100 μM, 25 μL) in the footpad. > 1100 nm. 65 mW/cm2. Scale bar: 1 cm. b. The normalized fluorescence intensity curve of (a) (n = 3, data were shown as means ± SD). Figure S4. Photostability of BSA@IR-780 in popliteal and sacral lymph nodes in vivo. a. Continuously irradiation of popliteal and sacral lymph nodes under 808 nm laser. The bilateral hind limbs were injected with BSA@IR-780 (300 μM, 25 μL) in the footpad. > 1100 nm. 65 mW/cm2. Scale bar: 1 cm. b. The normalized fluorescence intensity curve of (a) (n = 3, data were shown as means ± SD). Figure S5. Evaluation of blood circulation time of BSA@IR-780. a. NIR-II imaging of blood ex vivo post-injected of BSA@IR-780 (150 μM, 200 μL) via tail vein at different time points. b. The normalized quantitative curve of fluorescence intensity of blood at different time points (n = 3, data were shown as means ± SD). Figure S6. Representative NIR-II images of mesenteric LNs at different time points. The FE-2PEG was injected into the subserous layer of the cecum (300 μM, 20 μL). The injection site was massaged for 3 min to facilitate the flow of the probe in the lymphatic fluid. Over 1100 nm collection; 65 mW/cm2; Scale bar: 1 mm. Figure S7. The NIR-II imaging of mesenteric LNs at different time points. a. Representative NIR-II images of mesent [file 12951_2023_2171_MOESM1_ESM.docx]

**Additional file**

**High-precision Detection and Navigation Surgery of Colorectal Cancer Micrometastases**

*Shengjie Ma^a,b^, Bin Sun^b,c^, Mengfei Li^b,c^, Tianyang Han^b,c^, Chenlong Yu^b,c^, Xin Wang^b,c^, Xue Zheng^b,c^, Yinquan Zhao^a^, Shuang Li^a^, Shoujun Zhu^b,c,*^, Quan Wang^a,*^*

^a^ Department of Gastric and Colorectal Surgery, General Surgery Center, The First Hospital of Jilin University, Changchun, 130012, P.R. China

Email: [wquan@jlu.edu.cn](mailto:wquan@jlu.edu.cn)

^b^ Joint Laboratory of Opto-Functional Theranostics in Medicine and Chemistry, The First Hospital of Jilin University, Changchun, 130021, P.R. China

^c^ State Key Laboratory of Supramolecular Structure and Materials, College of Chemistry, Jilin University, Changchun 130012, P.R. China

Email: [sjzhu@jlu.edu.cn](mailto:sjzhu@jlu.edu.cn)

Materials and methods…………………………………………………….....Pages 3-13

Additional file figures……………………………………………………..…Pages 14-35

# Materials and methods

**Materials**

Ltd. PbCl_2_, CdO, anhydrous Na_2_SO_4_, Na_2_CO_3_, *N,N*-dimethylformamide (DMF), *N,N′*-dicyclohexylcarbodiimide, hexane, toluene, and poly (acrylic acid) (MW = ∼1800) were purchased from Aladdin. Sulfur powder was purchased from Alfa. IR-780 (≥98%), bovine serum albumin (BSA), oleylamine, oleic acid, 1-octadecene (ODE), and 2-(Nmorpholino) ethanesulfonic acid hydrate were purchased from Sigma-Aldrich. Short-pass filters (1300 nm) and long-pass filters (900, 1000, 1200, 1300, and 1500 nm) were purchased from Thorlabs. Cell Counting Kit-8 (CCK-8) was purchased from New Cell & Molecular Biotech Co., Ltd (China). Indocyanine green (ICG) (modified) for human injection was purchased from Dandong Yichuang Pharmaceutical Co. Ltd. D-Lucifcrin potassium salt was purchased from Beyotime Biotechnology Co. Ltd. The amphiphilic polymer oleylaminepoly (acrylic acid) (OPA) was kindly provided by the Joint Laboratory of Opto-Functional Theranostics in Medicine and Chemistry, Jilin University. All reagents were used as received without further purification. Deionized water was obtained from a Millipore water purification system.

**Characterization**

PerkinElmer Lambda 950 was employed to measure the ultraviolet-visible-near infrared (UV-VIS-NIR) absorption spectra. Fluorescent emission spectra were collected by an Edinburgh FL 920 spectrofluorometer equipped with 808 nm diode lasers.

**Preparation of FE-2PEG**

FE-2PEG was prepared following the previously reported method [1].

 **Scheme S1**. Synthesis of **IR-FE** and **IR-FE-N_3_**.

1. **Synthesis of IR-FE**

Compound 1 (4.9 g, 20 mmol) and compound 2 (97.6 g, 400 mmol) were added into the mixture of 40% NaOH (aq, 40 mL) and DMSO (40 mL), then the temperature was heated to 80 ℃ and stirred 4 h. The mixture was extracted, dried by MgSO_4_, and evaporated. Then pure compound 3 was separated successfully by column chromatography. Compound 4 (710 mg, 5 mmol) was added into dry tetrahydrofuran (THF) under N_2_ and *n*-BuLi (2.5 M, 2.4 mL) was slowly injected, then *n*-Bu_3_SnCl (3255 mg, 10 mmol) was injected and the whole process should be controlled at -78 ℃. Then compound 3 (844 mg, 1.5 mmol) and Pd(PPh_3_)_2_Cl_2_ (50 mg, 0.07 mmol) were added into the mixture. The temperature was heated to 120 ℃ and stirred overnight. The mixture was extracted, dried by MgSO_4_, and evaporated. Pure compound 7 was separated successfully by column chromatography. Compound 6 (590 mg, 0.93 mmol) was added into dry THF under N_2_ and *n*-BuLi (2.5 M, 0.75 mL) was slowly injected, then *n*-Bu_3_SnCl (608 mg, 1.87 mmol) was injected and the whole process should be controlled at -78 ℃. Then compound 8 (135 mg, 0.4 mmol) and Pd(PPh_3_)_2_Cl_2_ (70 mg, 0.1 mmol) were added into the mixture. The temperature was heated to 120 ℃ and stirred overnight through avoiding light. The mixture was extracted, dried by MgSO_4_, and evaporated. Then pure **IR-FE** was separated successfully as green solid (215 mg, 36.9%) by column chromatography.

1. **Synthesis of IR-FE-N_3_**

**IR-FE** (50 mg, 0.034 mmol) and KN_3_ (35 mg, 0.43 mmol) into DMF (6 mL), then the temperature was heated to 60 ℃ and stirred 12 h. The mixture was extracted, dried by MgSO_4_, and evaporated. Then pure **IR-FE-N_3_** was separated successfully as green solid (35 mg, 79.1%) by column chromatography. ^1^H NMR (400 MHz, Chloroform-d) δ 7.92 (d, J = 7.9 Hz, 2H), 7.78 – 7.66 (m, 6H), 7.40 – 7.27 (m, 6H), 4.60 – 4.31 (m, 8H), 3.13 (t, J = 7.0 Hz, 8H), 2.02 (dt, J = 11.0, 5.5 Hz, 8H), 1.47 – 1.35 (m, 8H), 1.13 (pd, J = 9.4, 4.5 Hz, 16H), 0.66 (h, J = 7.9 Hz, 8H). ^13^C NMR (126 MHz, Chloroform-d) δ 152.58, 150.75, 150.59, 141.92, 140.79, 140.32, 138.31, 131.71, 127.14, 126.93, 125.58, 122.70, 120.61, 119.87, 119.76, 113.11, 108.73, 64.70, 64.54, 60.34, 55.06, 51.33, 40.21, 37.39, 37.06, 31.89, 30.00, 29.67, 29.38, 28.63, 27.06, 26.26, 23.54, 22.66, 21.00, 19.70, 14.16. MALDI-TOF-MS calcd for C_68_H_70_N_16_O_4_S_4_, M_w_ = 1302.46, Found 1301.457.

1. **Synthesis of FE-2PEG**

A solution of IR-FE-N_3_ (35 mg, 0.027 mmol) in 3 mL of anhydrous THF was added alkylnyl-PEG600-OH (0.136 mmol), tris[(1-benzyl-1H-1,2,3-triazol-4-yl)methyl]amine (0.40 mmol) and copper(I) thiophene-2-carboxylate (0.40 mmol) respectively. The mixture was stirred for 10 min at r.t. under the N_2_ atmosphere. Subsequently, this solution was filtered, evaporated, and purified column chromatography to remove the excess PEG. The crude product was further dialyzed for three days and lyophilized to afford FE-2PEG as green oils.

**Preparation of BSA@IR-780**

IR-780 was mixed with BSA in a 1:1 molar ratio and shaken for two hours at 50°C using a shaker to obtain BSA@IR-780 [2].

**Preparation of QDs**

PbS@CdS quantum dots (QDs) were synthesized according to the reported protocol [3, 4]. The sulfur precursor solution was prepared by mixing 0.08 g (5 mmol) of sulfur powder and 7.5 mL of oleylamine in a two-neck flask at 120 °C under argon for 30 min. The lead precursor solution was prepared by mixing 0.834 g (3 mmol) of PbCl_2_ and 7.5 mL of oleylamine in a three-neck flask and degassing for 30 min under argon at 120 °C and then increased to 160 °C. 2.25 mL of the sulfur precursor solution (0.75 mmol of S) was rapidly injected into the Pb precursor solution (3 mmol of Pb) under stirring. The temperature was maintained at 160 °C throughout the reaction. After 30 min, the reaction was quenched by adding 10 mL of cold hexane and 20 mL of ethanol. The products were collected by centrifugation and re-suspended in 10 mL of hexane. The mixture was agitated for 10 min to remove excess sulfur from the products. The PbS QDs were precipitated via centrifugation. This precipitation procedure with oleic acid was repeated three times until the supernatant was colorless. After centrifugation of the suspension and decantation of the supernatant, the PbS QDs were re-suspended in 12 mL of toluene and 3 mL of ODE. PbS@CdS QDs were synthesized via the cation-exchange procedure. CdO (1.2 g, 9.2 mmol), oleic acid (8 mL), and ODE (20 mL) were heated to 200 °C, purged with argon, and then cooled down to 100 °C. A 5 mL portion of the previously prepared PbS QDs suspended in ODE was bubbled with argon for 10 min and then added to the Cd precursor solution. The reaction flask was quenched with 5 mL of cold hexane after the reaction was maintained at 100 °C for 30 min. PbS@CdS QDs were precipitated with ethanol and then re-dispersed in hexane. PbS@CdS QDs (5.0 mg) were dissolved in 2.0 mL of chloroform containing 15 mg of OPA. The mixture was stirred at room temperature for 30 min, and the solvent was removed under vacuum by a rotary evaporator. The residue was then dissolved in 2 mL of 50 mM Na_2_CO_3_ solution under sonication. PbS@CdS QDs were collected by ultracentrifuge at 50,000 rpm for 1 h, and washed three times with deionized water.

**Cell lines and culture**

CT-26-Luc cells were kindly provided by the Changchun Institute of Applied Chemistry Chinese Academy of Sciences. L-929 cells were kindly provided by the Joint Laboratory of Opto-Functional Theranostics in Medicine and Chemistry, Jilin University. CT-26-Luc cells were cultured in RPMI-1640 with 10% fetal bovine serum (FBS) and 1% penicillin/streptomycin (P/S) and incubated at 37°C with 5% CO_2_. L929 cells were cultured in DMEM containing 10% FBS and 1% P/S at 37°C under an atmosphere of 5% CO_2_.

**Cell toxicity measurement**

The in vitro cytotoxicity of FE-2PEG was investigated by the CCK-8 method. Briefly, CT-26-Luc cells and L-929 cells were seeded in 96-well plates (5×10^3^ cells per well) and incubated for 24 h at 37 °C in a humidified incubator with 5% CO_2_. The RPMI-1640 medium was removed, and fresh PBS was used to wash the adherent CT-26-Luc and L-929 cells in triplicate. Then, the fresh RPMI-1640 medium containing FE-2PEG (0, 1, 5, 10, and 20 μM) was added and subsequently incubated for 12 h and 24 h. 100 μL of fresh RPMI-1640 medium containing 10 μL CCK-8 solution was used to replace RPMI-1640 containing FE-2PEG and followed by co-incubation for an additional 1 h. After incubation, the absorbance value of each well was measured at 450 nm by an Elisa reader (Bio-Tek, Synergy LX, USA) to evaluate the cytotoxicity of FE-2PEG.

**H&E staining**

After being harvested, the tumor, lymph nodes, peritoneal nodules, biopsies, heart, liver, spleen, lung, and kidney were fixed in 4% paraformaldehyde overnight. These tissues were dehydrated, embedded in paraffin, and sectioned into 3 μm thick slides. H&E staining was then performed according to the protocol of the H&E kit (Beyotime Institute of Biotechnology, Cat. No. C0105). H&E staining images of all tissues were acquired by the upright Nikon Eclipse 80i microscope.

**Animals and tumor model**

All animal experiments were conducted under the institutional guidelines and were approved by the Experimental Animal Ethical Committee of The First Hospital of Jilin University (Protocol number: 20210642). BALB/c, C57BL/6 mice were purchased from the Charles River Co. Lt. Bedding, nesting materials, food, and water were provided ad libitum. Ambient temperature was controlled at 20-24 °C with 12-hour light/12-hour dark cycles. Before the experiment, all mice were shaved using Nair hair removal lotion and anesthetized with tribromoethanol or isoflurane.

To establish the subcutaneous tumor model of colon cancer, CT-26-Luc cells (2×10^6^ cells in 50 μL PBS) were injected subcutaneously into the left hindlimb. For the orthotopic colorectal cancer (CRC) model, 2x10^6^ CT-26-Luc cells suspension was injected into the subserosa layer of the cecum with the microliter syringe (100 μL, Shanghai Bolige Industry & Trade Co., Ltd). The needle was slowly moved forward about 1 cm from the terminal to the mesenteric side of the cecum, aiming to avoid injuring blood vessels. Sterile distilled water was dropped on the cecum to kill spilled tumor cells aming to avoid intraperitoneal dissemination. The orthotopic CRC model was successfully established after two weeks for further experiments. Peritoneal carcinomatosis was induced by intraperitoneal injection of 2x10^6^ CT-26-Luc cells, then monitored for one week for further experiments.

**Bioluminescence imaging**

The growth of tumor models was monitored by bioluminescence imaging (BLI) with the IVIS Lumina III (PerkinElmer) system. Generally, the D-Luciferin potassium salt was dissolved in sterile D-PBS, as a 15 mg/mL solution, and the solution was filtered using a 0.2 μm membrane for the removal of bacteria. The D-Luciferin was injected intraperitoneally into each mouse at a dose of 10 μL/g. BLI analysis was performed 10-20 min post the D-luciferin injection.

**Biosafety evaluation study**

Six- to eight-week-old female BALB/c mice were randomly grouped. FE-2PEG, ICG, and BSA@IR-780 were administered once with the dose of 300 μL (300 μM), 200 μL (150 μM), and 200 μL (150 μM) via tail vein injection (n = 3 per group). The mice were sacrificed, and the organs (e.g., hearts, livers, spleens, lungs, and kidneys) were harvested for hematoxylin-eosin (H&E) staining at 30 days. The body weight of the mice was measured at an interval of 48 hours up to two weeks.

Blood samples from BALB/c mice were collected after intravenous administration of FE-2PEG, ICG, and BSA@IR-780 (300 μL (300 μM), 200 μL (150 μM), and 200 μL (150 μM), respectively) at 30 days (n = 3, per group). Blood samples were tested for liver function, kidney function, and routine blood tests. Liver and kidney function tests include: alanine aminotransferase (ALT), aspartate aminotransferase (AST), total bilirubin (TBIL), albumin (ALB), alkaline phosphatase (ALP), γ-glutamyltranspeptidase (γ-GT), total bile acid (TBA), urea (UREA), creatinine (CREA), and uric acid (UA). Routine blood tests include: white blood cell (WBC), red blood cell (RBC), hemoglobin (HGB), hematokrit (HCT), mean corpuscular volume (MCV), mean corpuscular hemoglobin (MCH), mean corpuscular hemoglobin concentration (MCHC), red blood cell distribution width (RDW), platelet (PLT), mean platelet volume (MPV), and the distribution width of platelets (PDW).

**Intraoperative navigation surgery of subcutaneous and orthotopic tumors**

CT-26-Luc tumor-bearing mice were injected with FE-2PEG via the tail vein. The NIR-II imaging was performed with the condition: 808 nm laser excitation, ~65 mW/cm^2^ power density. All NIR-II images were collected on a two-dimensional InGaAs camera. All mice were anesthetized and experienced fluorescence-guided surgery (FGS) at 24 hours (n = 3 per group). After removing the tumor, the resected area was re-examined under the fluorescence navigation system, and the residual tumor was removed. The corresponding fluorescence intensity was analyzed using ImageJ software.

**Intraoperative navigation surgery of CRC peritoneal metastases**

Peritoneal metastasis mice were intravenously injected with FE-2PEG to evaluate the potential capability of NIR-II FGS for tumors at the millimeter level. Peritoneal metastases were removed under NIR-II FGS at 24 hours, then re-examined and biopsied in the diaphragm, mesocolon, mesentery, omentum, and Douglas pouch with naked eyes. Resected nodules were all examined with H&E staining to verify the efficacy of NIR-II FGS.

**NIR-Ⅱ microscopic imaging**

Frozen tissue sections (5 μm) of the primary tumor and lymph nodes were performed. Two adjacent slices of the tumor’s largest length section were used, one of which was microscopically imaged under the NIR-II camera. The other was examined with H&E staining to confirm the tumor cells' presence and the cut edges' status.

**Statistical analysis**

Images’ process and quantification of the fluorescence signal were both performed by using ImageJ software (1.8.0, National Institutes of Health, USA). Graphs were generated by Graphpad Prism (Graphpad Prism 8.0.2, Graphpad Software) and Origin (Origin 2019b, Origin Software). The experimental data were expressed as mean ± standard deviation (SD), and the Student’s t-test was conducted to assess the differences between different groups (*P < 0.05 considered significant, *, **, and *** represent P < 0.05, P < 0.01, and P < 0.001, respectively). The apparent width of the corresponding capillary was calculated using the Gaussian fitting full-width half maximum (FWHM) by the Origin software (Origin 2019b, OriginLab).

# Additional file figures


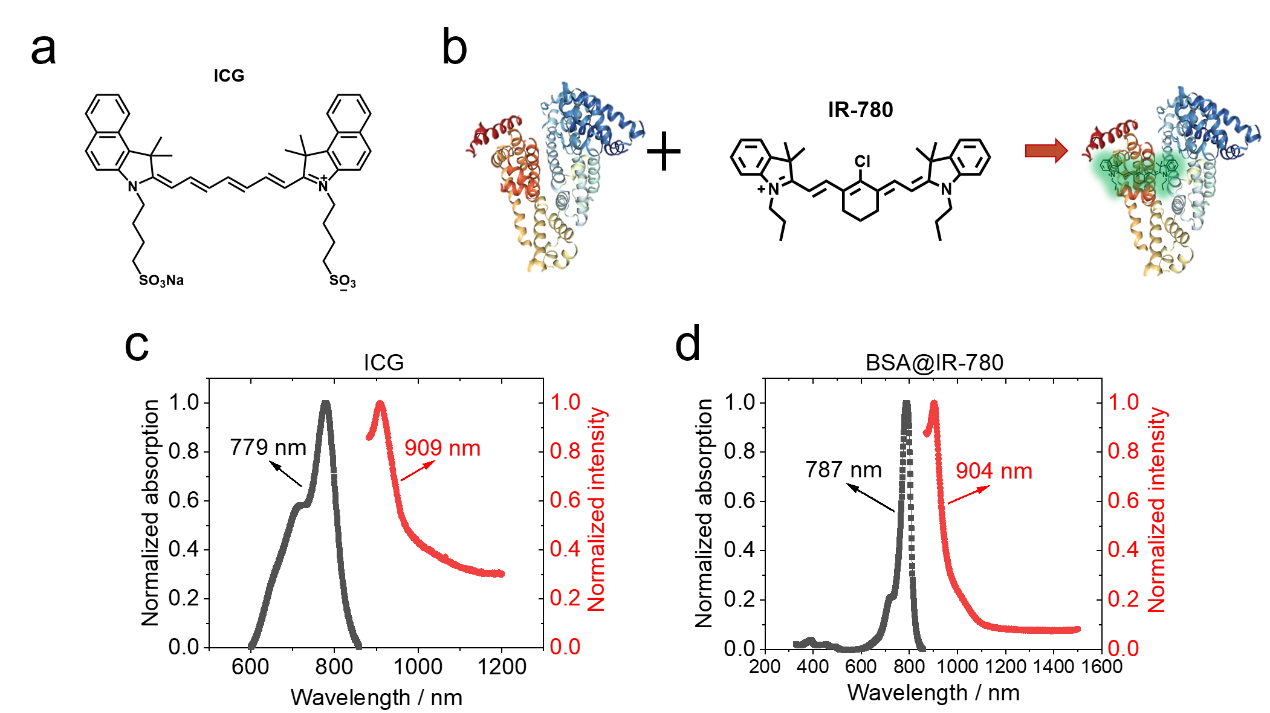


Figure S1. Characterization of ICG and BSA@IR-780. a. Schematic diagram of molecular structure of ICG. b. Schematic diagram of the molecular structure for BSA@IR-780. c. Absorption and emission spectra of ICG. d. Absorption and emission spectra of BSA@IR780.


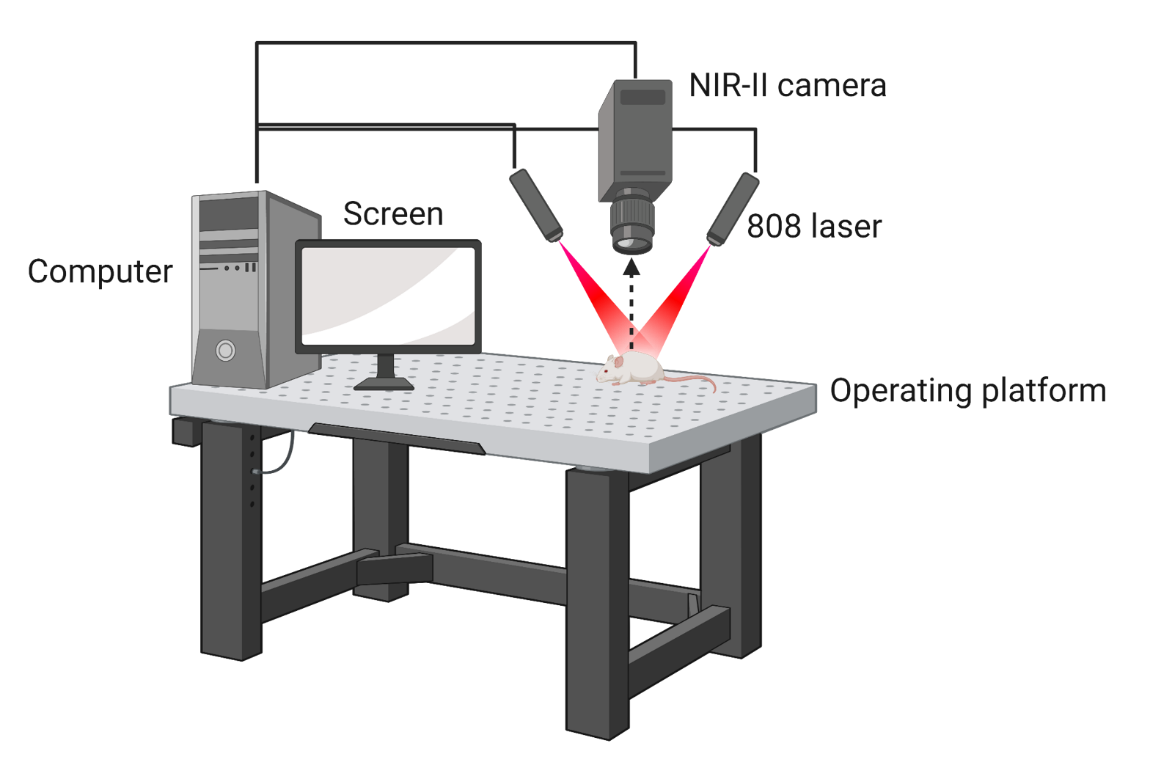


Figure S2. The scheme of the NIR-II imaging and operating platform. Created with BioRender.com.


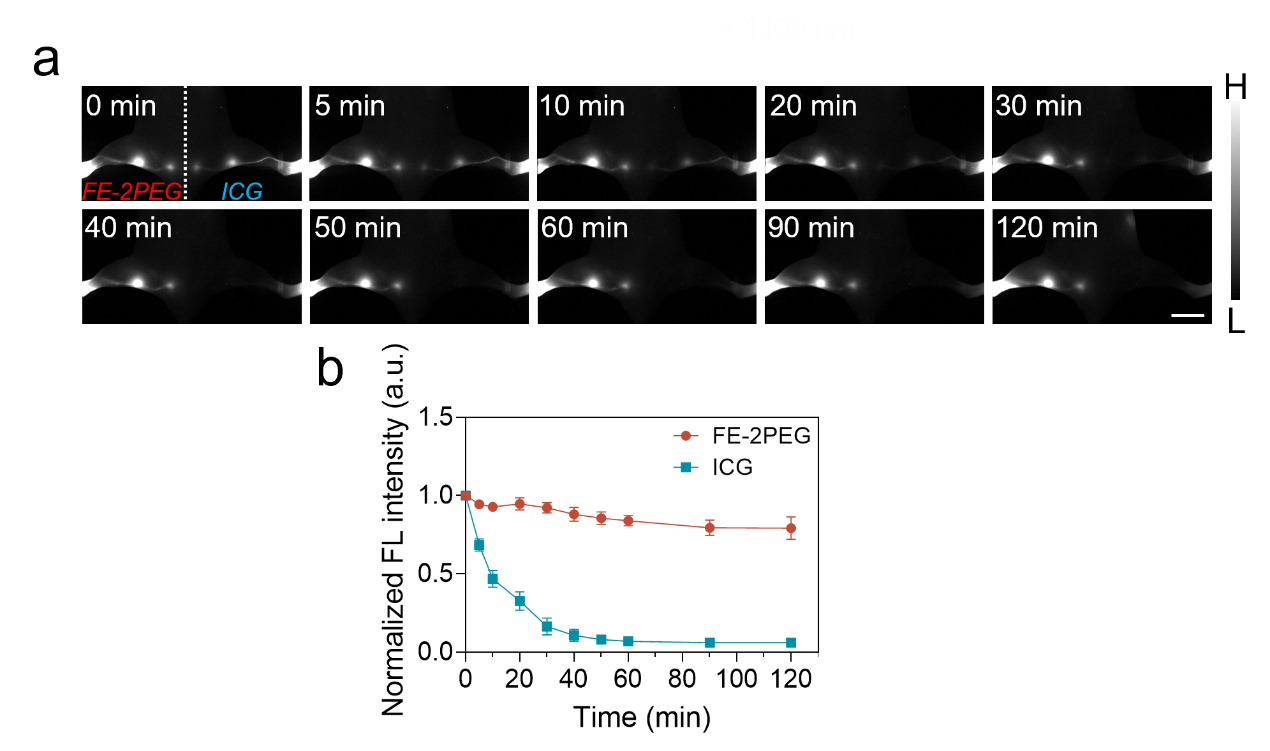


Figure S3. Photostability of FE-2PEG and ICG in popliteal and sacral lymph nodes. a. Continuously irradiation of popliteal and sacral lymph nodes under 808 nm laser. The left hind limb was injected with FE-2PEG (600 μM, 25 μL), and the right hind limb was injected with ICG (100 μM, 25 μL) in the footpad. > 1100 nm. 65 mW/cm^2^. Scale bar: 1 cm. b. The normalized fluorescence intensity curve of (a) (n = 3, data were shown as means ± SD).


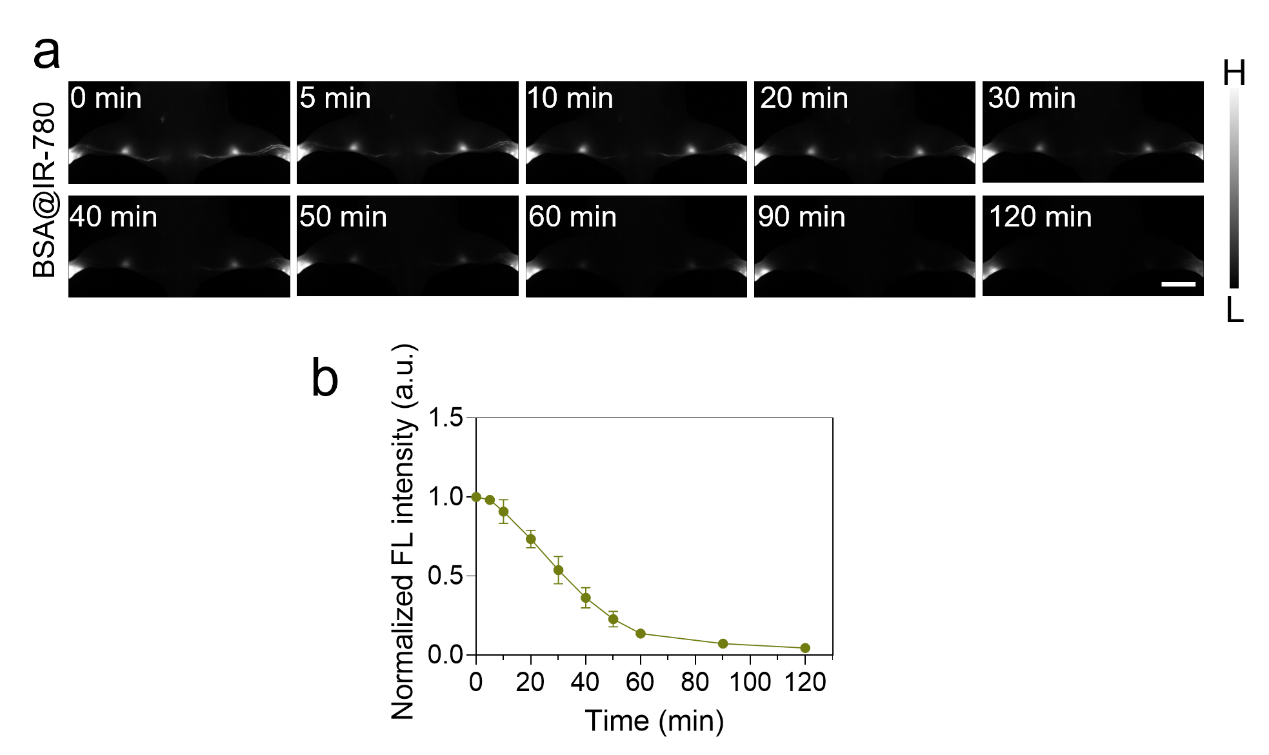


Figure S4. Photostability of BSA@IR-780 in popliteal and sacral lymph nodes in vivo. a. Continuously irradiation of popliteal and sacral lymph nodes under 808 nm laser. The bilateral hind limbs were injected with BSA@IR-780 (300 μM, 25 μL) in the footpad. > 1100 nm. 65 mW/cm^2^. Scale bar: 1 cm. b. The normalized fluorescence intensity curve of (a) (n = 3, data were shown as means ± SD).


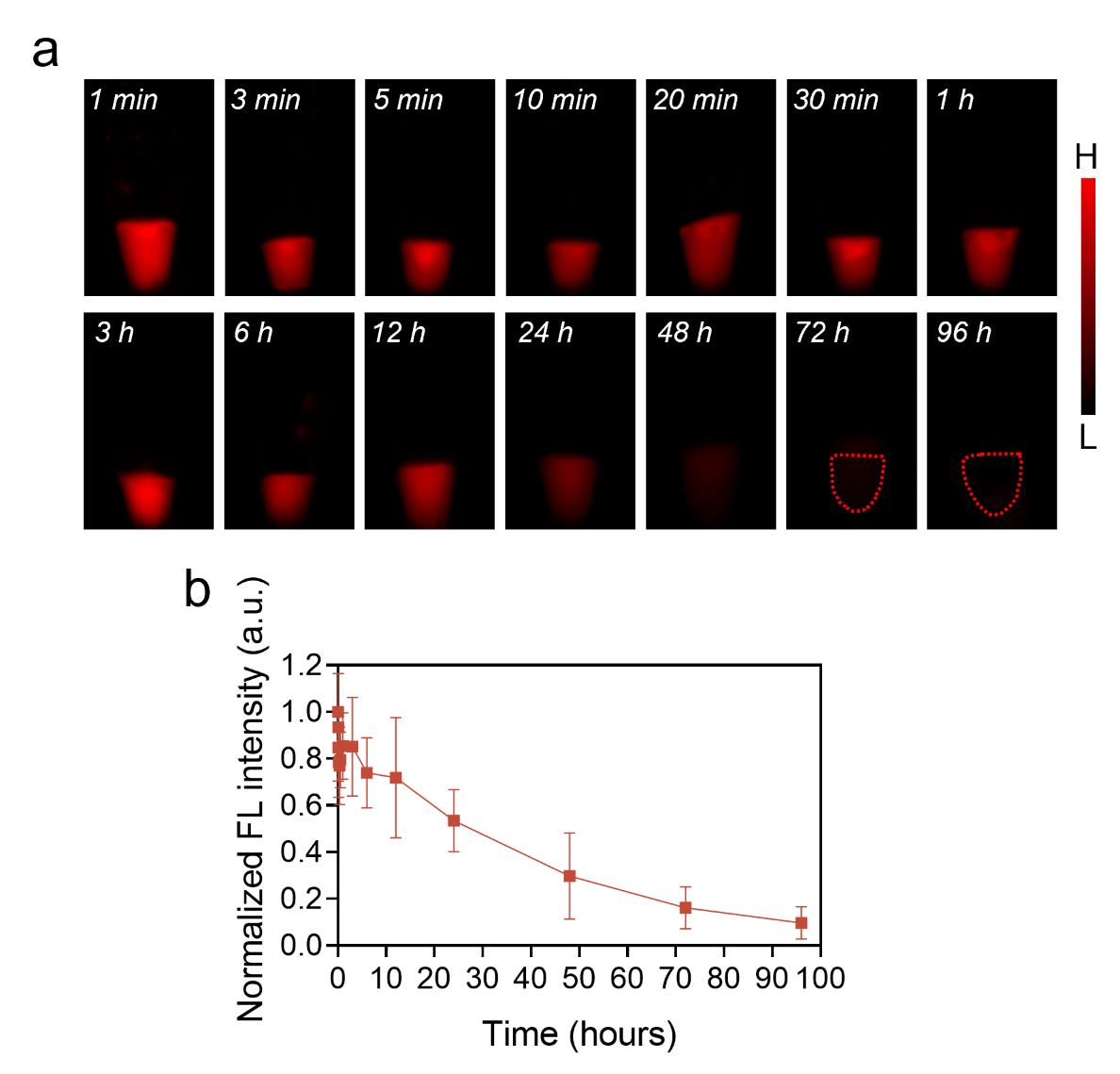


Figure S5. Evaluation of blood circulation time of BSA@IR-780. a. NIR-Ⅱ imaging of blood ex vivo post-injected of BSA@IR-780 (150 μM, 200 μL) via tail vein at different time points. b. The normalized quantitative curve of fluorescence intensity of blood at different time points (n = 3, data were shown as means ± SD).


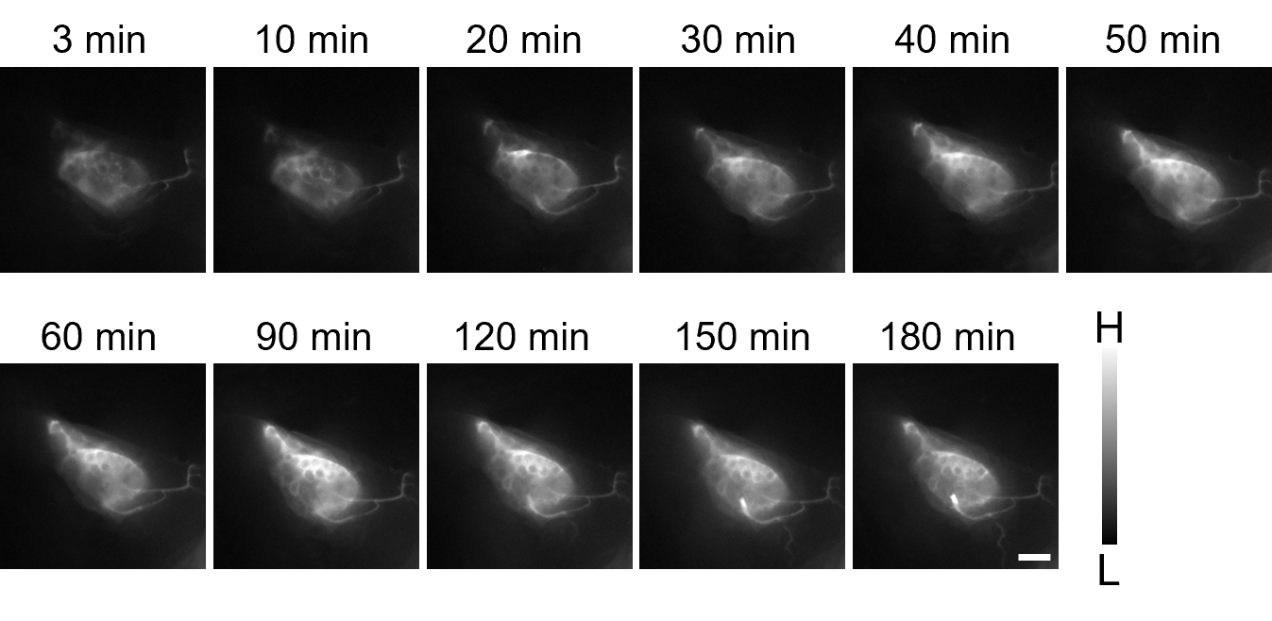


Figure S6. Representative NIR-II images of mesenteric LNs at different time points. The FE-2PEG was injected into the subserous layer of the cecum (300 μM, 20 μL). The injection site was massaged for 3 min to facilitate the flow of the probe in the lymphatic fluid. Over 1100 nm collection; 65 mW/cm^2^; Scale bar: 1 mm.


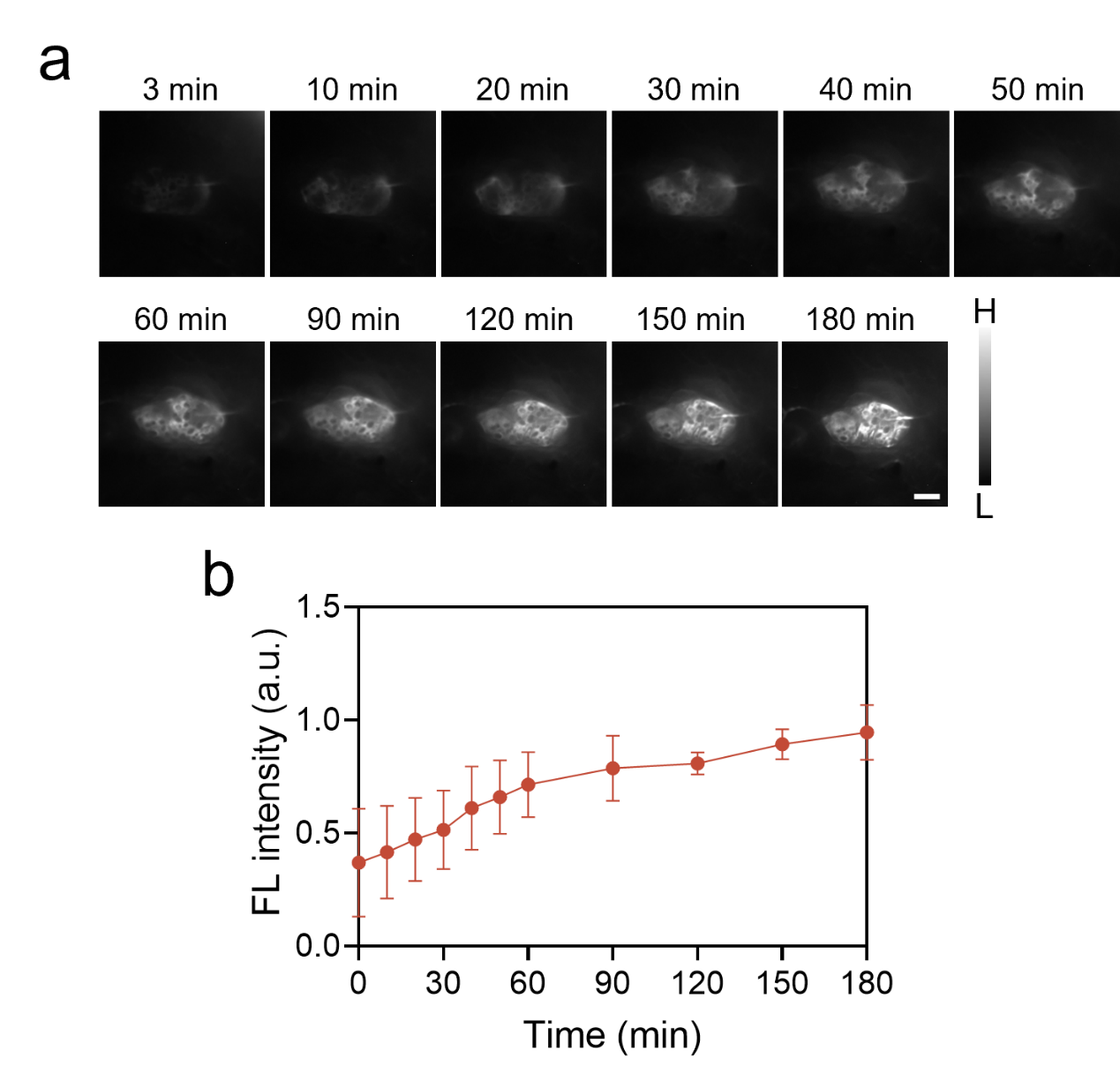


Figure S7. The NIR-II imaging of mesenteric LNs at different time points. a. Representative NIR-II images of mesenteric LNs. The probe FE-2PEG was injected into the subserous layer of the cecum (300 μM, 20 μL). The injection site was massaged for 3 min at the start, and then intermittently massaged for 30 seconds before each capture. b. The normalized fluorescent intensity curve of mesenteric LNs (n=5, data were shown as mean ± SD). Over 1100 nm collection; 65 mW/cm^2^; Scale bar: 1 mm.


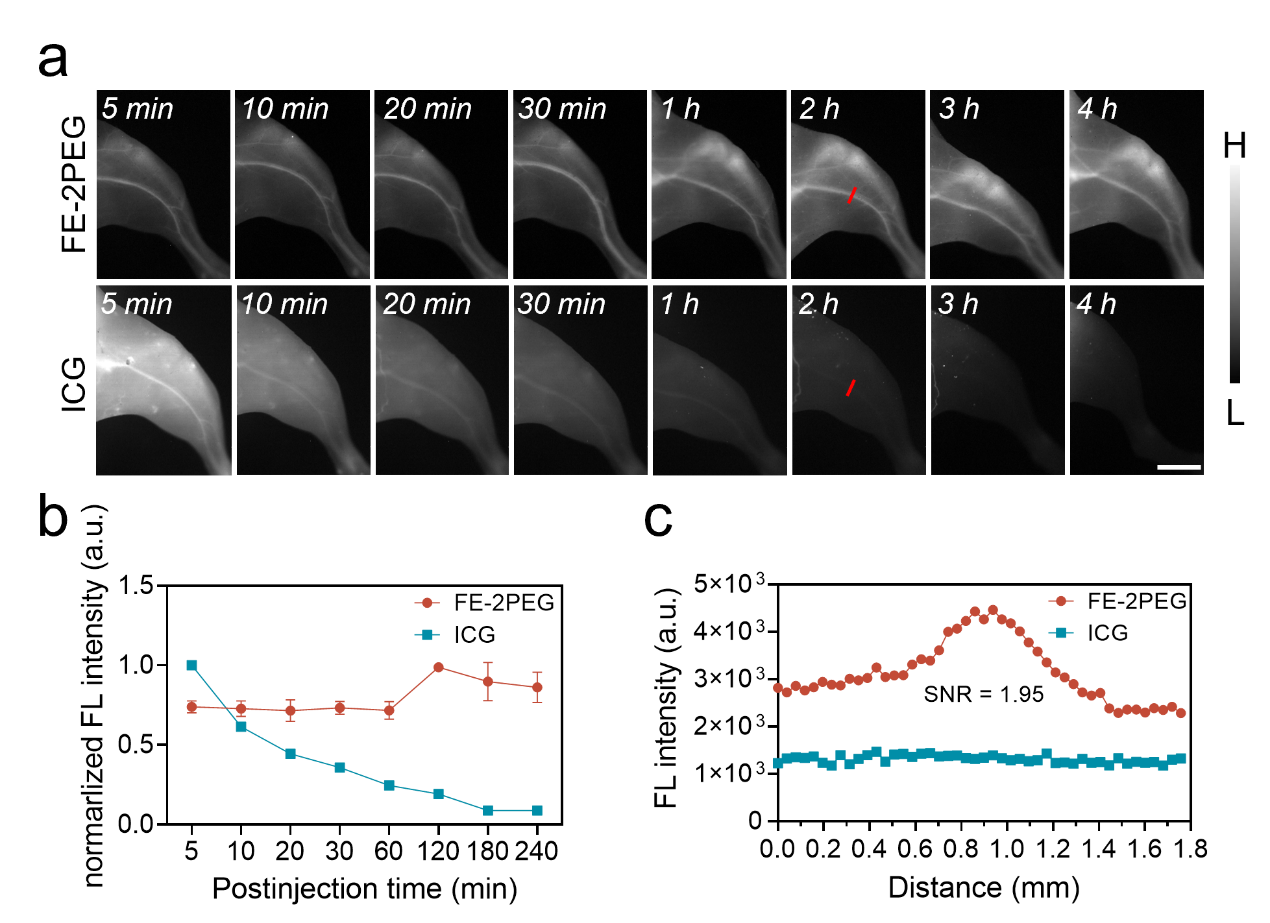


Figure S8. NIR-II imaging of FE-2PEG and ICG in blood vessels of the hindlimb. a. Representative NIR-II imaging of FE-2PEG and ICG in blood vessels of the hindlimb at different time points. Scale bar: 5 mm. b. Normalized FL intensity in vessels of FE-2PEG and ICG. c. Plot profiles of lines on blood vessels of hindlimb at 2 hours (n = 3, data were shown as means ± SD).


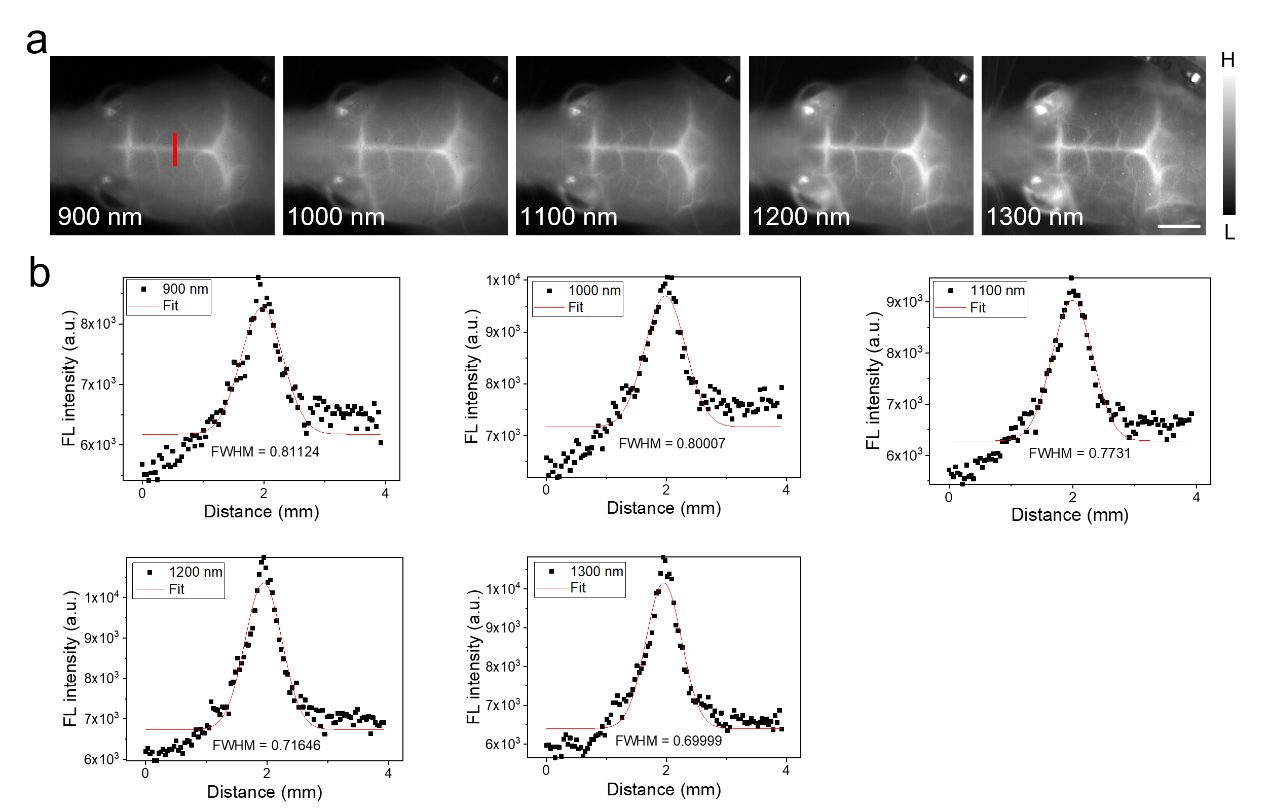


Figure S9. The wavelength-dependence NIR-II imaging of FE-2PEG in cerebral vessels. a. Representative images of the cerebral vessels in NIR-II (900 nm, 1000 nm, 1100 nm, 1200 nm, and 1300 nm) windows. Scale bar: 5 mm. b. Fluorescence cross-sectional intensity distribution of cerebral vessels in different NIR-II windows (red line in (a)) (n = 3). The full-width half maxima (FWHM) are 0.81124, 0.80007, 0.7731, 0.71646, and 0.69999 for 900 nm, 1000 nm, 1100 nm, 1200 nm, and 1300 nm, respectively.


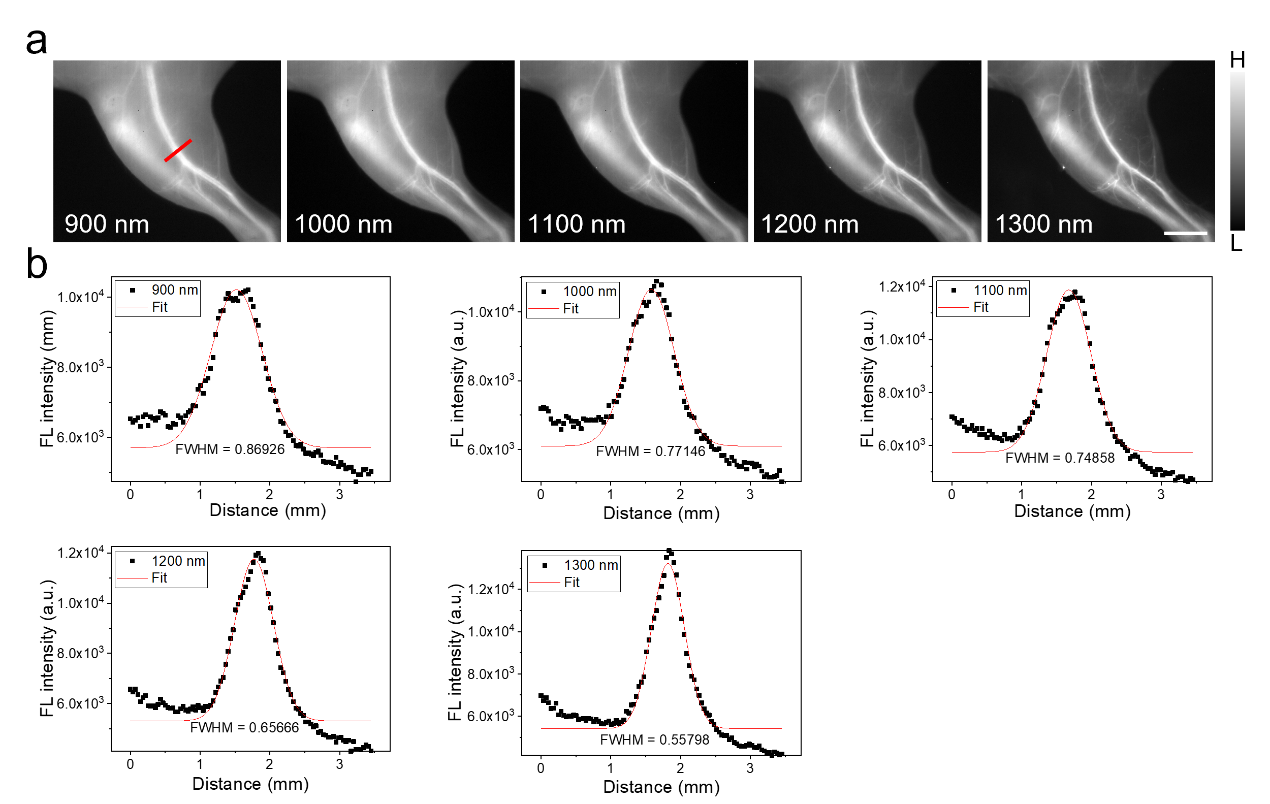


Figure S10. The wavelength-dependence NIR-II imaging of FE-2PEG in blood vessels of the hindlimb. a. Representative images of blood vessels in the hindlimb in NIR-II (900 nm, 1000 nm, 1100 nm, 1200 nm, and 1300 nm) windows. Scale bar: 5 mm. b. Fluorescence cross-sectional intensity distribution of blood vessels in hindlimb in different NIR-II windows (red line in (a)) (n = 3). The full-width half maxima (FWHM) are 0.86926, 0.77146, 0.74858, 0.65666, and 0.55798 for 900 nm, 1000 nm, 1100 nm, 1200 nm, and 1300 nm, respectively.


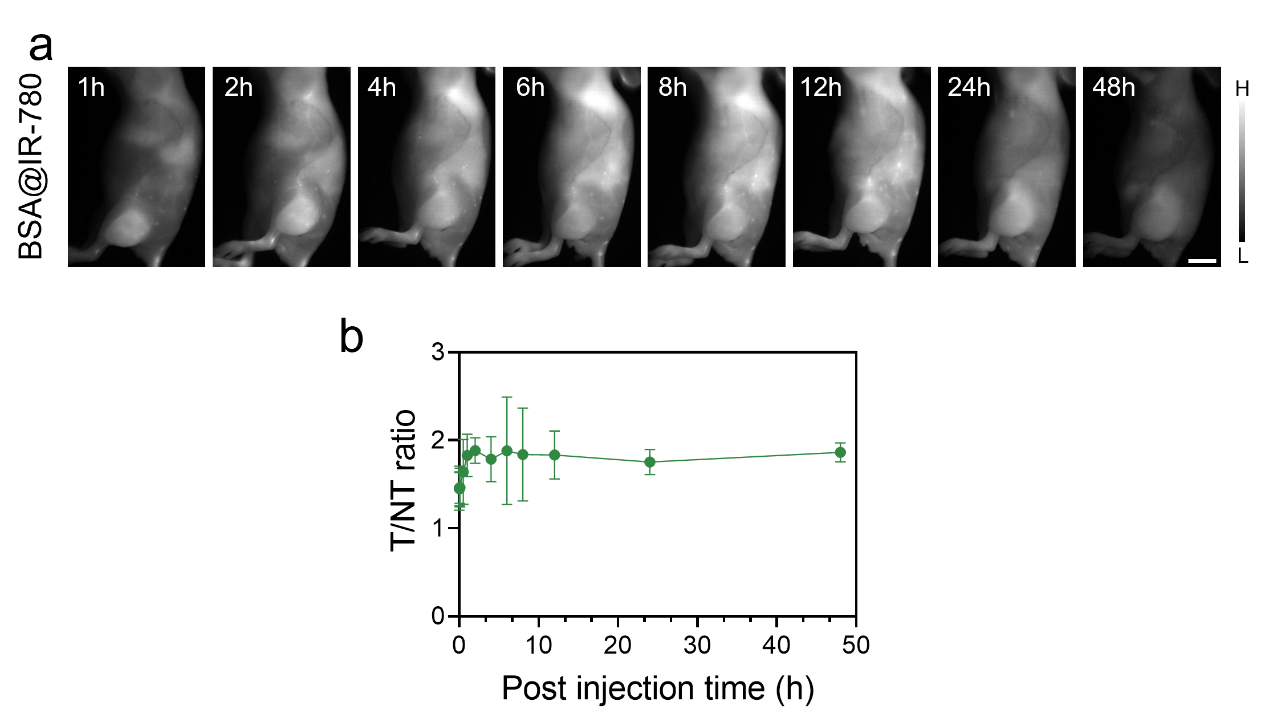


Figure S11. NIR-II in vivo imaging of BSA@IR-780 in the subcutaneous tumor. a. Representative NIR-II in vivo images of BSA@IR-780 (150 μM, 200 μL) in subcutaneous tumor at different time points. Lateral position. Tail vein injection. Scale bar: 1 cm. b. The T/NT ratio of subcutaneous tumors at different time points in vivo.


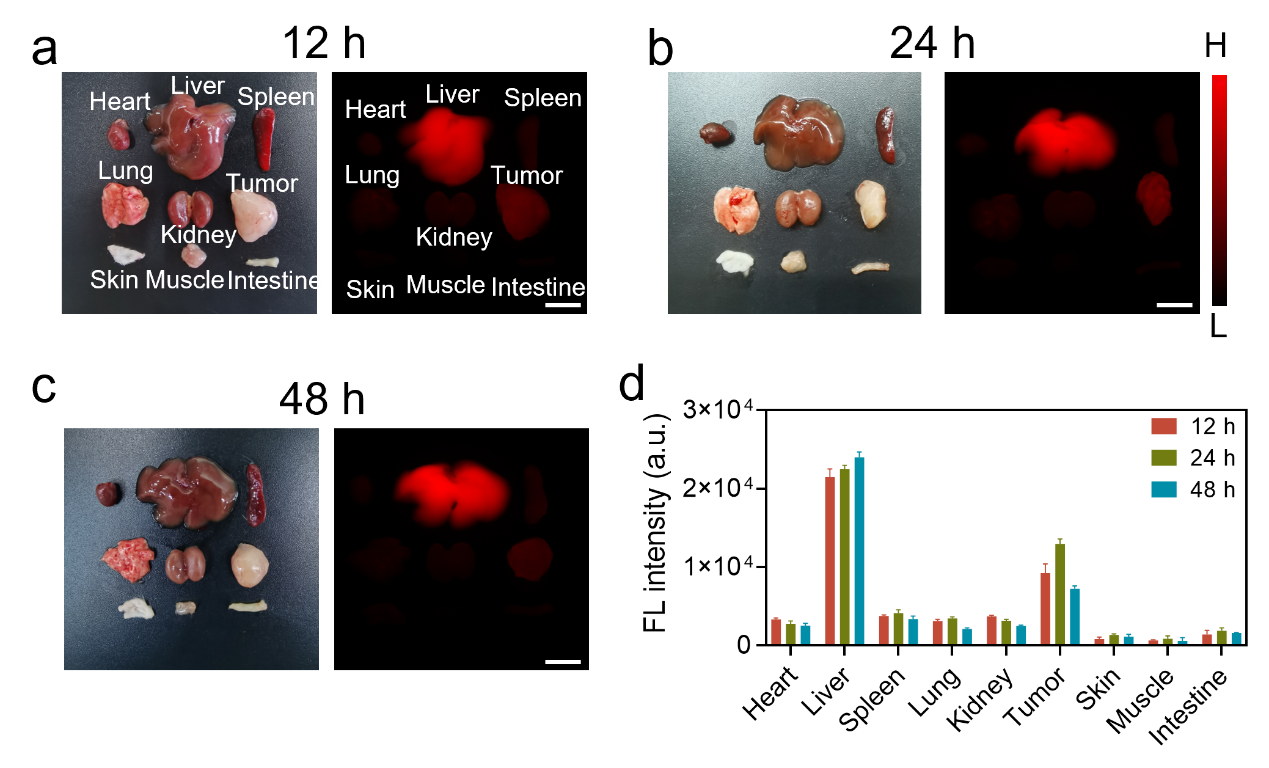


Figure S12. Evaluation of the biodistribution of FE-2PEG. a. White light and NIR-II imaging of the tumor, organs (heart, liver, spleen, lung, kidney, intestine), and tissues (skin, muscle) at 12h. Scale bar: 1 cm. b. White light and NIR-II imaging of the tumor, organs, and tissues at 24h. Scale bar: 1 cm. c. White light and NIR-II imaging of the tumor, organs, and tissues at 48h. Scale bar: 1 cm. d. Quantitative analysis of fluorescence intensity signals of tumors, organs, and tissues at different time points (n = 3, data were shown as means ± SD).


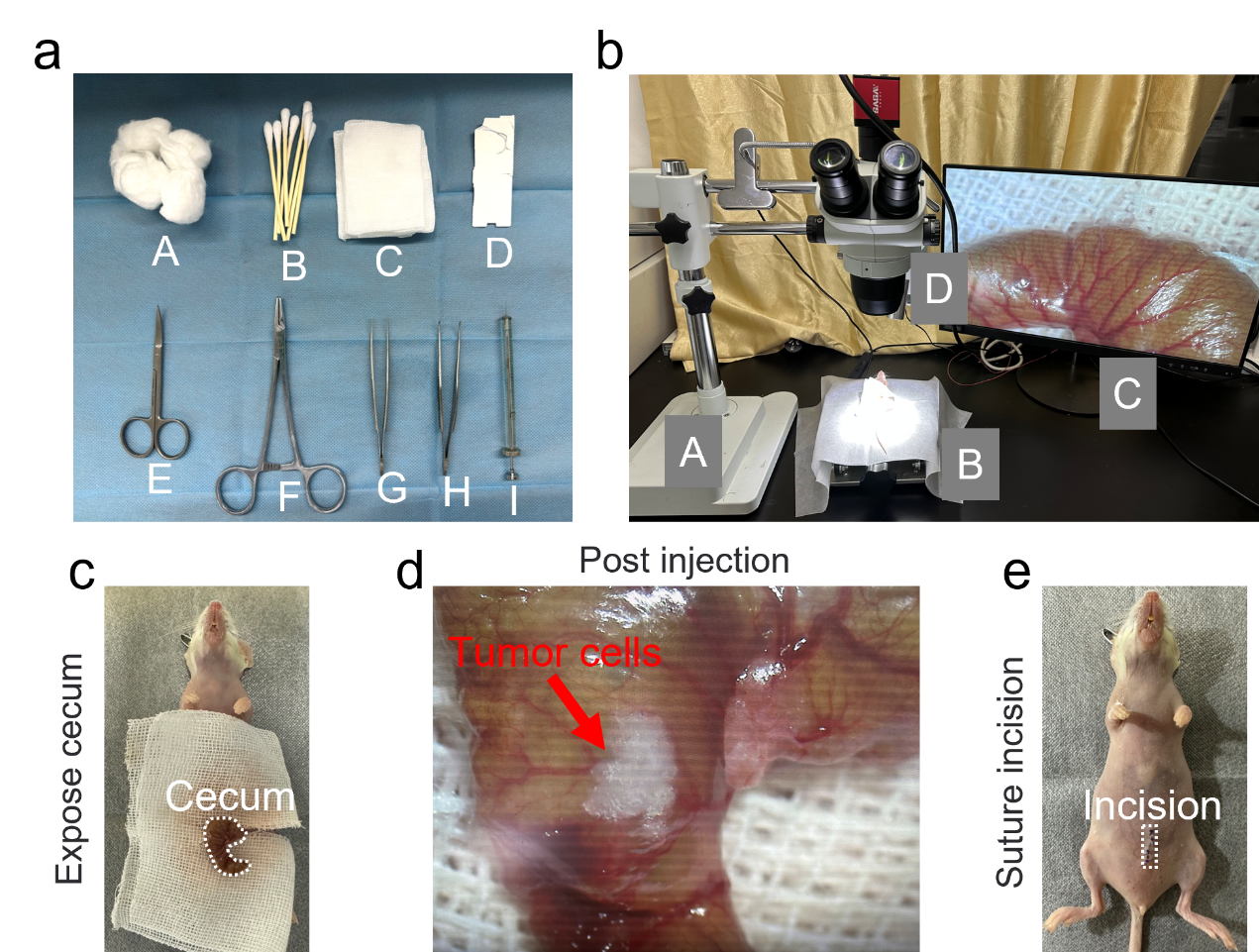


Figure S13. The establishment of the orthotopic mouse model of CRC. a. The preparation of surgical instruments and materials. Materials for surgery include Cotton (A), cotton swabs (B), gauze (C), and absorbable sutures (D). Instruments for surgery include bent scissor (E), needle holder (F), straight tweezer (G), bent tweezer (H), and micro-syringe (I). b. The surgical operating platform includes the stereomicroscope (A), operating table (B), monitor (C), and LED lamp (D). c. The cecum was exposed through an abdominal incision. The dotted line shows the cecum. The gauze was used to protect the cecum. d. The CT-26-Luc cells were injected into the subserous membrane of the cecum with the micro-syringe. The red arrow shows tumor cells. e. The incisions in the abdomen were sutured layer by layer with absorbable sutures. The dotted line shows the abdominal incision after the suture.


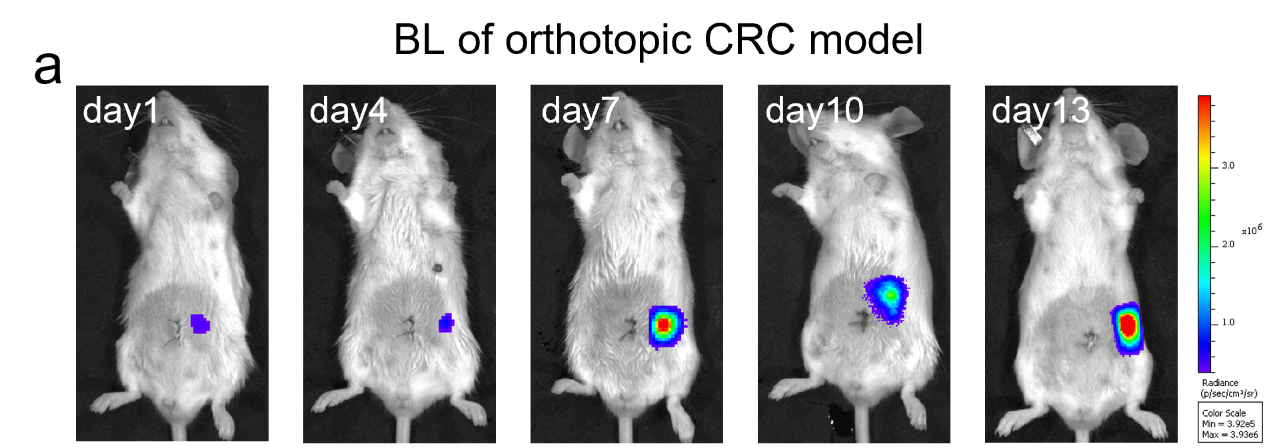


Figure S14. BLI monitoring of the orthotopic CRC model formation.


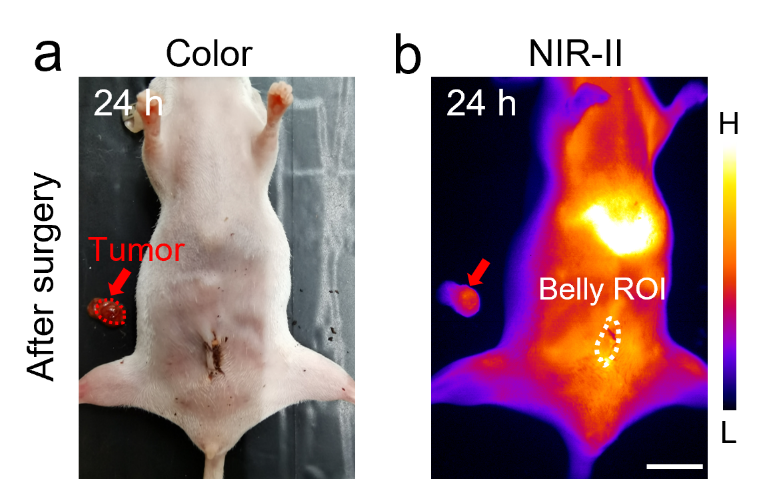


Figure S15. The NIR-II imaging of post-operative orthotopic CRC mice. a. The photograph of post-operative orthotopic CRC mice. b. Representative NIR-II imaging of post-operative orthotopic CRC mice post-injected with FE-2PEG via the tail vein at 24 hours. Scale bar: 1 cm.


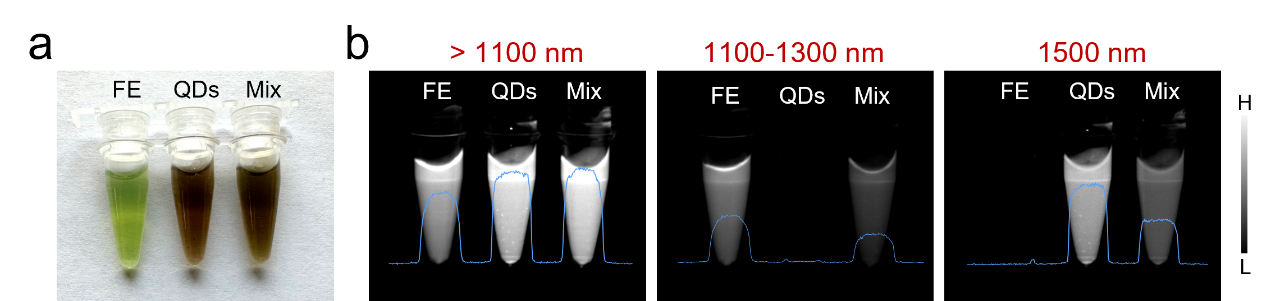


Figure S16. Dual-NIR-II color testing of FE-2PEG, QDs, and mixture in vitro. a. The photograph of the FE-2PEG, QDs, and mixture solutions. b. The NIR-II fluorescent signals were collected under > 1100, 1100 ~ 1300, and > 1500 nm windows, respectively.


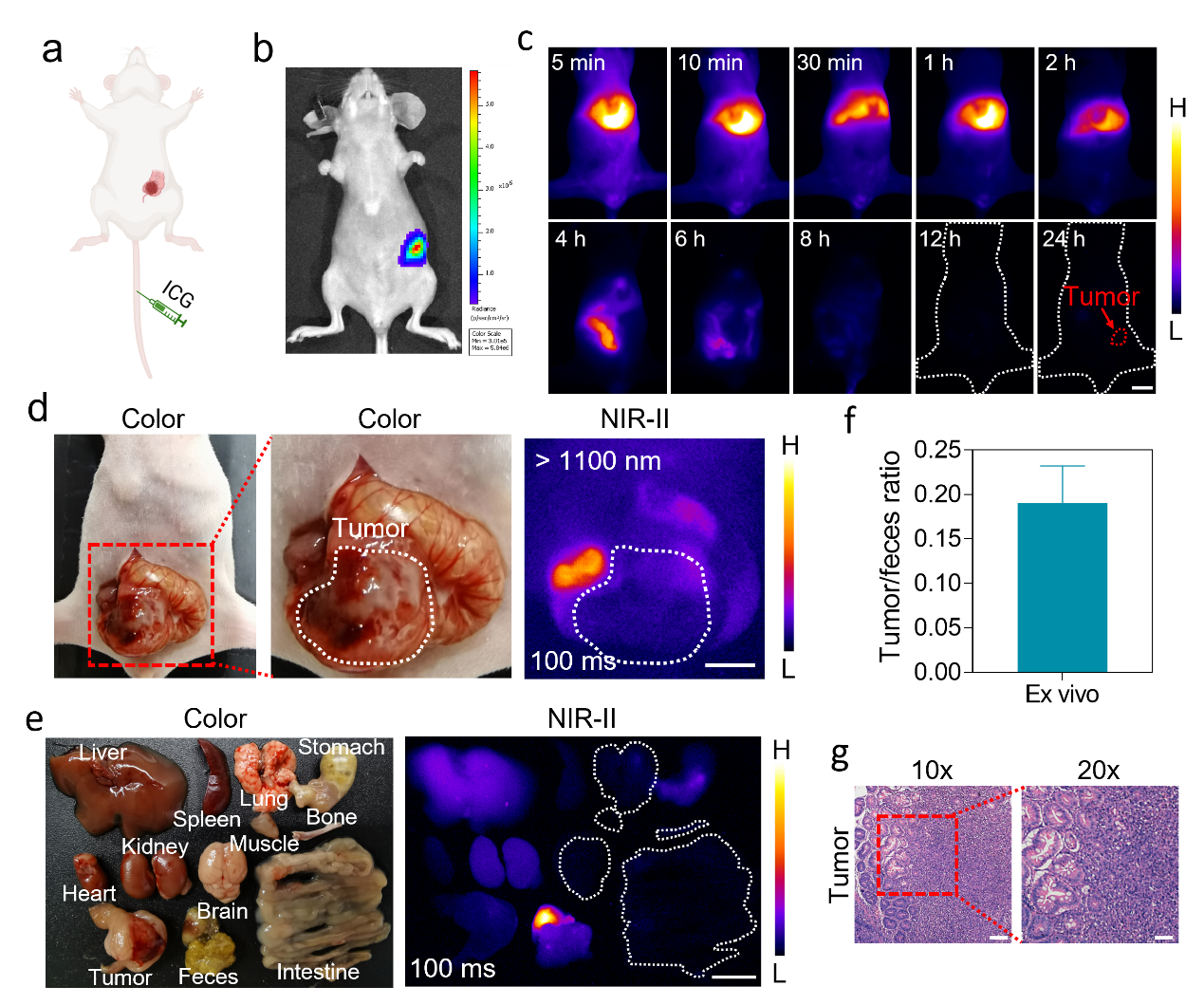


Figure S17. NIR-II imaging of ICG in orthotopic CRC in vivo. a. The schematic diagram showed that the orthotopic CRC mouse was injected with ICG (150 μM, 200 μL) via the tail vein. b. BLI showed the successful establishment of the orthotopic CRC model. c. Representative images of orthotopic CRC post-injected ICG at different time points in vivo. Scale bar: 1 cm. d. White light and NIR-II imaging of exposed orthotopic CRC in vivo. > 1100 nm. Exposure time = 100 ms. Scale bar: 5 mm. e. White light and NIR-II imaging of the organs and tumor ex vivo > 1100 nm, exposure time = 100 ms. Scale bar: 1 cm. f. The fluorescence intensity ratio of tumor to feces ex vivo. (n = 3, data were shown as means ± SD). g. H&E staining of the orthotopic tumor. Scale bar: 100 μm (10x); Scale bar: 50 μm (20x).


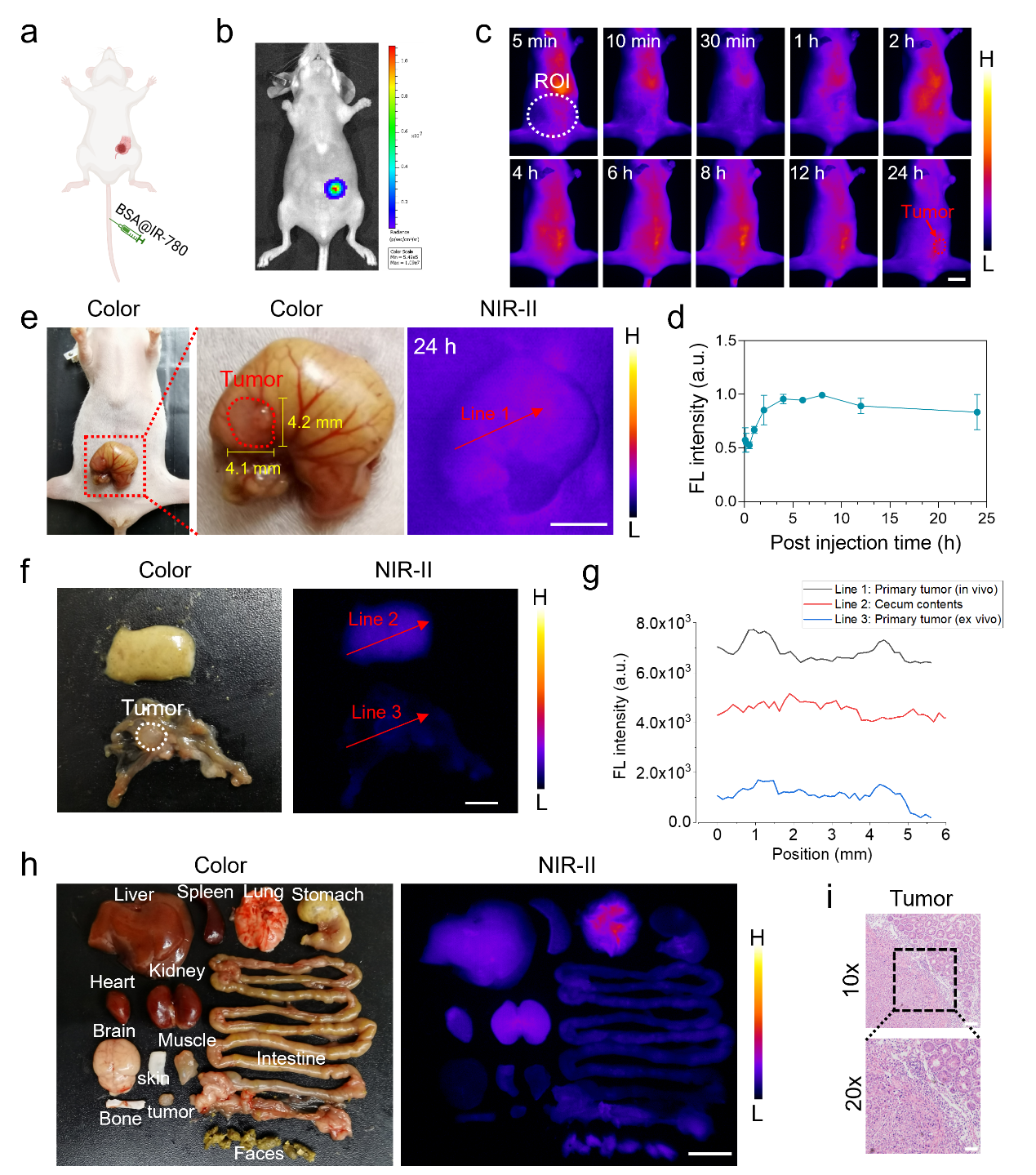


Figure S18. NIR-II imaging of BSA@IR-780 in orthotopic CRC. a. The schematic diagram showed that the orthotopic CRC mouse was injected with BSA@IR-780 (150 μM, 200 μL) via the tail vein. b. BLI showed the successful establishment of the orthotopic CRC model. c. Representative NIR-II images of BSA@IR-780 in orthotopic CRC at different time points with a supine position. Scale bar: 1 cm. d. Quantitative intensity signal analysis of abdominal ROI. e. White light and NIR-II imaging of the exposed orthotopic tumor in vivo. The tumor size is 4.2 mm in length and 4.1 mm in width. Scale bar: 5 mm. f. White light and NIR-II imaging of the isolated tumor and feces ex vivo. Scale bar: 5 mm. g. Plot profiles quantitative analysis of the tumor in vivo (line 1), the isolated feces (line 2), and the tumor ex vivo (line 3). h. White light and NIR-II imaging of isolated organs, feces, and the tumor ex vivo. Scale bar: 1 cm. i. H&E staining of the orthotopic tumor. Scale bar: 100 μm (10x); Scale bar: 50 μm (20x).


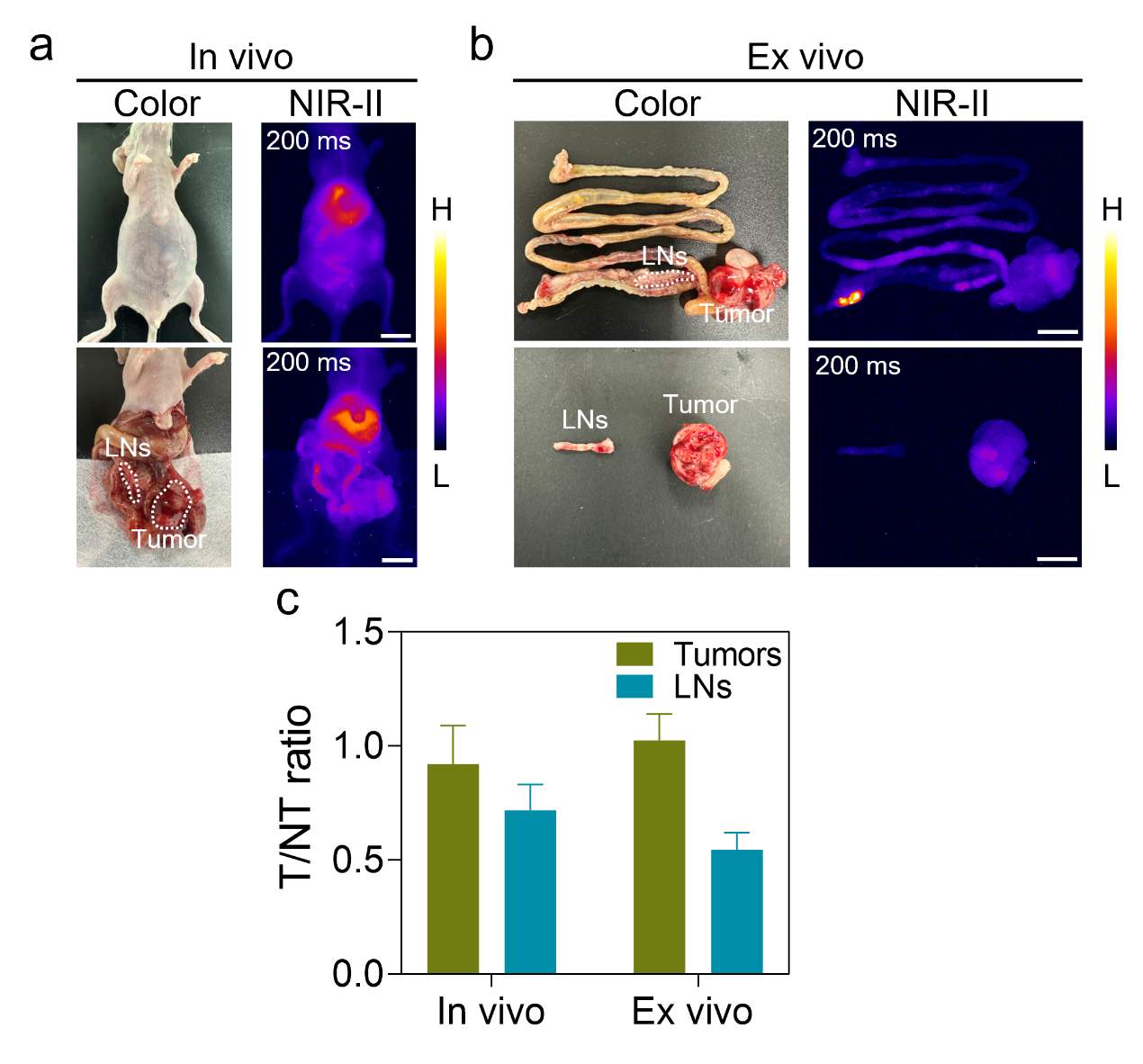


Figure S19. NIR-II imaging of ICG in orthotopic CRC with lymphatic metastases. a. White light and NIR-II imaging of orthotopic tumor and metastatic LNs before and after laparotomy in vivo. Scale bar: 1 cm. b. White light and NIR-II imaging of intestinal canal, orthotopic tumor, and metastatic LNs ex vivo. Scale bar: 1 cm. c. T/NT ratio of the orthotopic tumor and metastatic LNs in vivo and ex vivo (n = 3, data were shown as means ± SD).


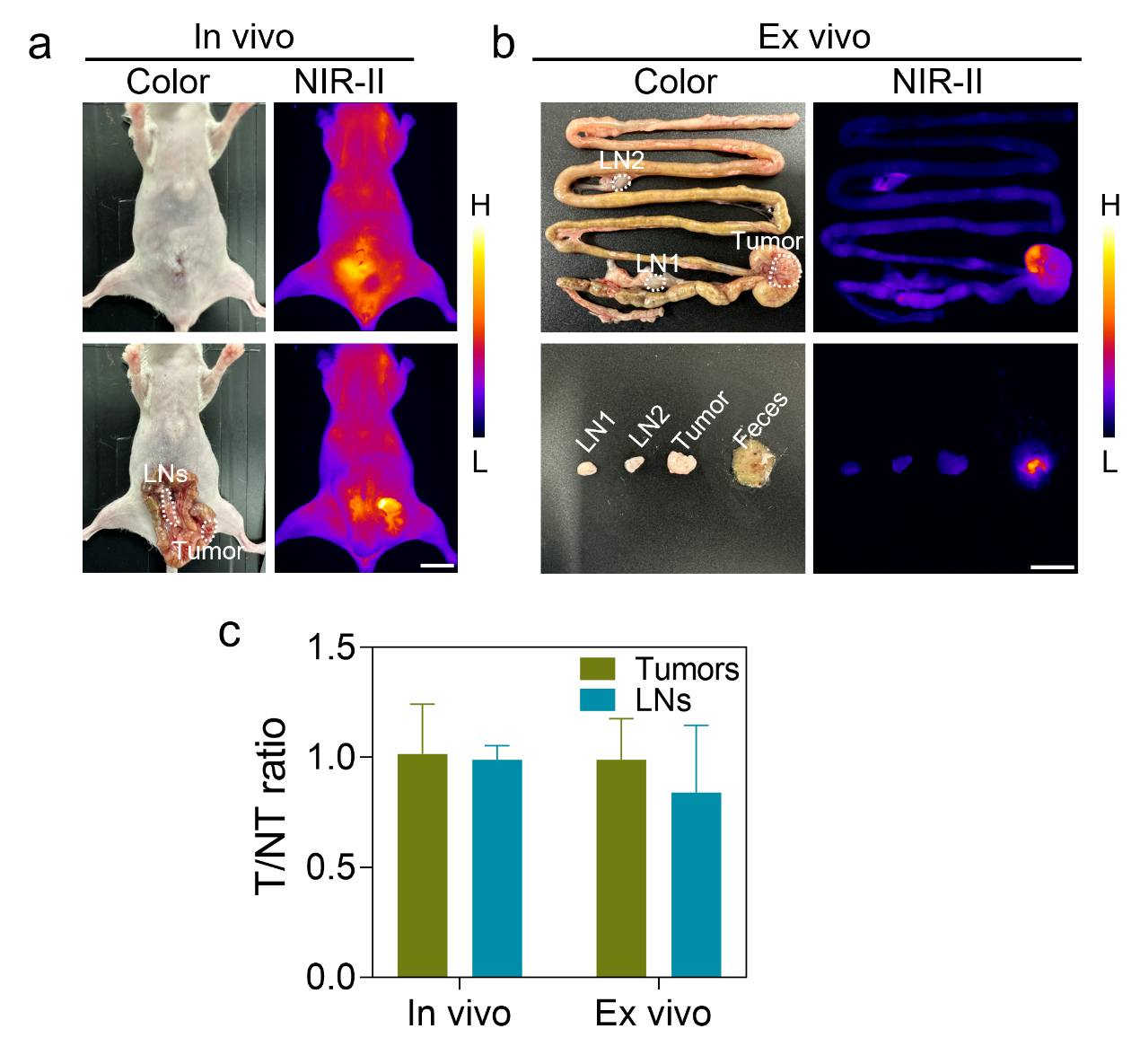


Figure S20. NIR-II imaging of BSA@IR-780 of orthotopic CRC with lymphatic metastases. a. White light and NIR-II imaging of primary tumor and metastatic lymph nodes displayed before and after laparotomy in vivo. Scale bar: 1 cm. b. White light and NIR-II imaging of intestinal canal, orthotopic tumor, metastatic LNs, and feces ex vivo. Scale bar: 1 cm. c. T/NT ratio of the orthotopic tumor and metastatic LNs in vivo and ex vivo.


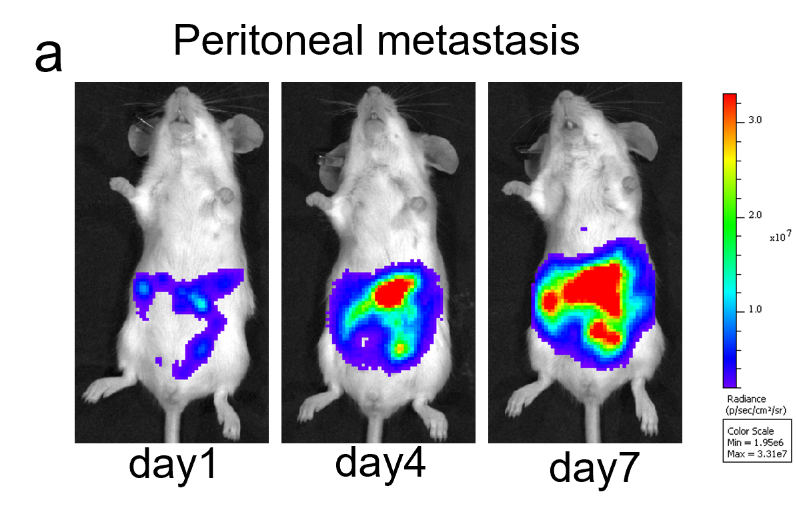


Figure S21. BLI of the establishment of CRC peritoneal metastasis model.


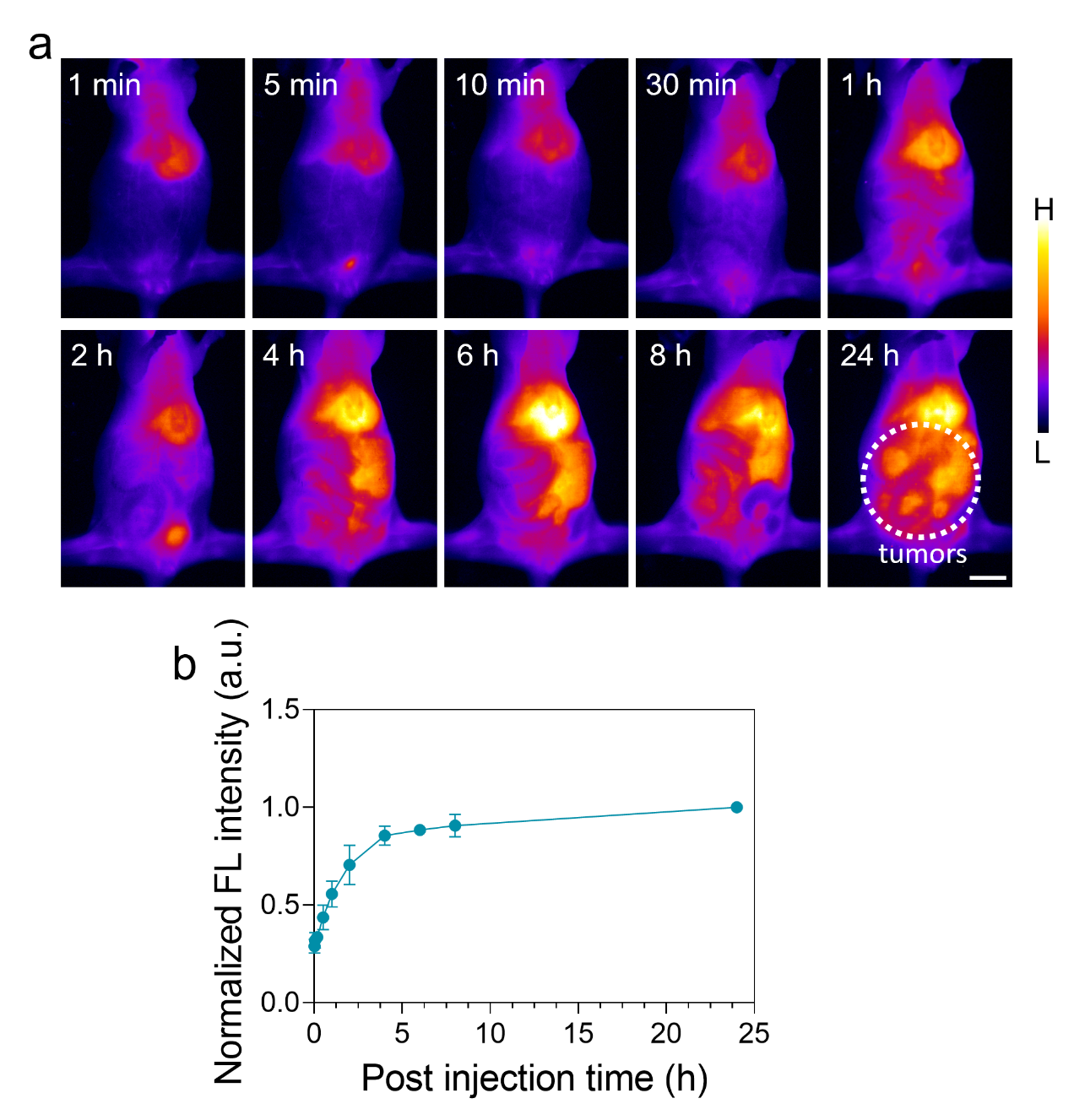


Figure S22. NIR-II in vivo imaging of FE-2PEG in CRC peritoneal metastasis model. a. Representative NIR-II images of FE-2PEG in peritoneal metastasis mice at different time points. Supine position, tail vein injection, 300 μM, 300 μL. Scale bar: 1 cm. b. Quantitative signal intensity analysis of abdominal ROI.


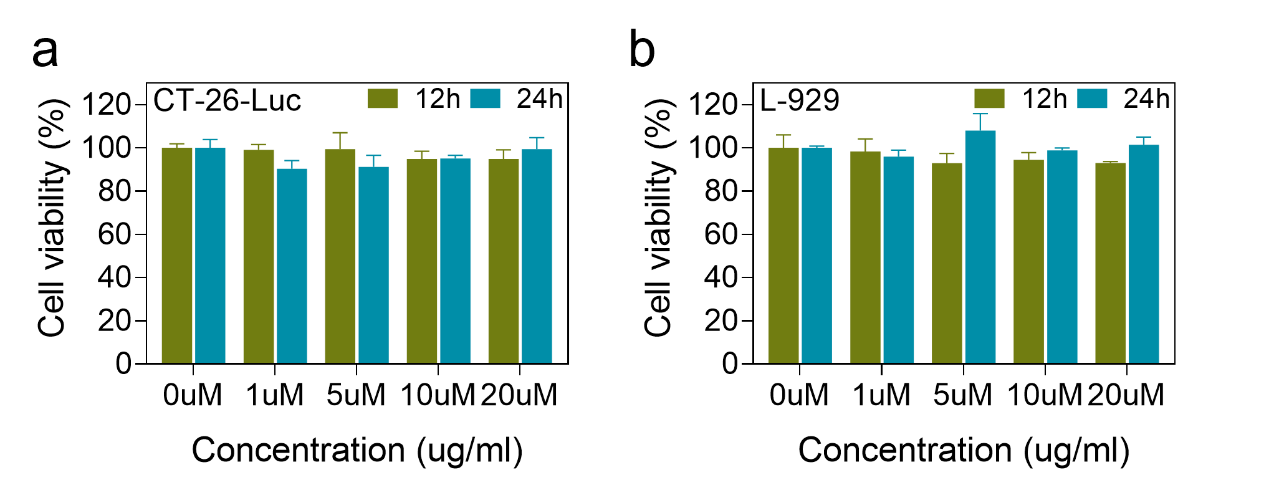


Figure S23. Biological safety of FE-2PEG. a. CCK-8 viabilities of CT-26-Luc cells after incubated with various concentrations of FE-2PEG for 12 h and 24 h; b. Viabilities of L-929 cells after incubation with various concentrations of FE-2PEG for 12 h and 24 h. (n = 6, data were shown as means ± SD).


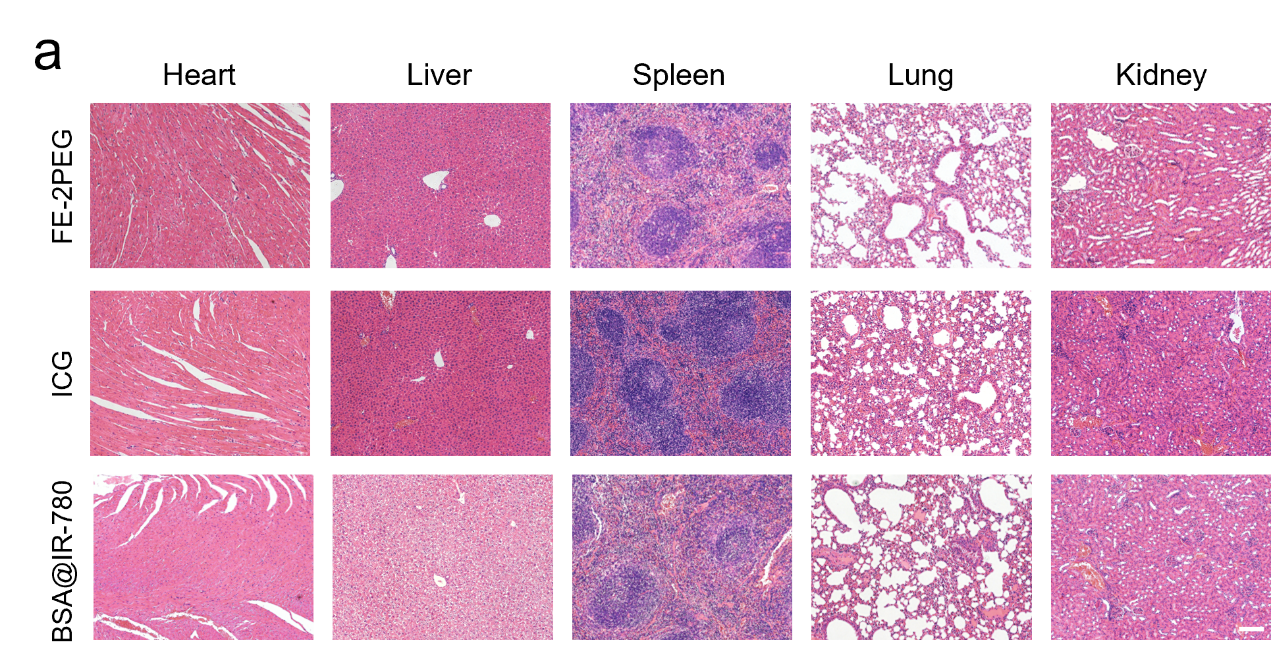


Figure S24. Biological safety of FE-2PEG, ICG, and BSA@IR-780. a. H&E staining of organs (Heart, liver, spleen, lung, and kidney) from mice after intravenous injection of FE-2PEG (300 μM, 300 μL), ICG (150 μM, 200 μL), and BSA@IR-780 (150 μM, 200 μL) in 30 days (n = 3). Scale bar: 100 μm.


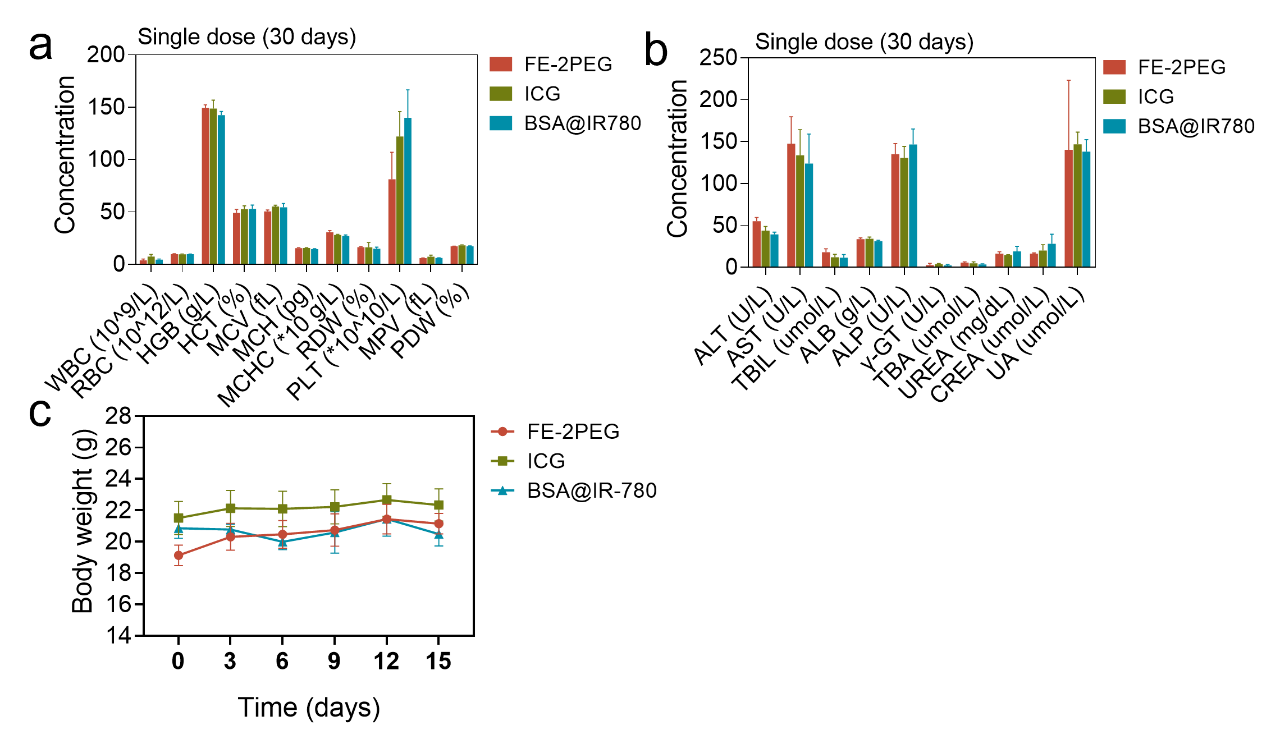


Figure S25. Biological safety of FE-2PEG, ICG, and BSA@IR780. a. Blood routine examination in 30 days post-injected of FE-2PEG (300 μM, 300 μL), ICG (200 μM, 150 μL), and BSA@IR-780 (200 μM, 150 μL) via tail vein. (n = 3, data were shown as means ± SD). b. Liver and renal function examination in 30 days post injection of FE-2PEG (300 μM, 300 μL), ICG (200 μM, 150 μL), and BSA@IR-780 (200 μM, 150 μL) via tail vein. (n = 3, data were shown as means ± SD). c. Body weight change in two weeks post-injected of FE-2PEG (300 μM, 300 μL), ICG (200 μM, 150 μL), and BSA@IR-780 (200 μM, 150 μL) via tail vein. (n = 3, data were shown as means ± SD).

**Additional file references**

1. Yang Q, Ma Z, Wang H, Zhou B, Zhu S, Zhong Y, Wang J, Wan H, Antaris A, Ma R, et al: Rational Design of Molecular Fluorophores for Biological Imaging in the NIR-II Window**.** *Adv Mater* 2017, 29.

2. Xu J, Han T, Wang Y, Zhang F, Li M, Bai L, Wang X, Sun B, Wang X, Du J, et al: Ultrabright Renal-Clearable Cyanine-Protein Nanoprobes for High-Quality NIR-II Angiography and Lymphography**.** *Nano Lett* 2022, 22**:**7965-7975.

3. Zhang M, Yue J, Cui R, Ma Z, Wan H, Wang F, Zhu S, Zhou Y, Kuang Y, Zhong Y, et al: Bright quantum dots emitting at ∼1,600 nm in the NIR-IIb window for deep tissue fluorescence imaging**.** *Proc Natl Acad Sci U S A* 2018, 115**:**6590-6595.

4. Tian R, Ma H, Zhu S, Lau J, Ma R, Liu Y, Lin L, Chandra S, Wang S, Zhu X, et al: Multiplexed NIR-II Probes for Lymph Node-Invaded Cancer Detection and Imaging-Guided Surgery**.** *Adv Mater* 2020, 32**:**e1907365.
